# Most chromatin interactions are not in linkage disequilibrium

**DOI:** 10.1101/gr.238022.118

**Published:** 2019-03

**Authors:** Sean Whalen, Katherine S. Pollard

**Affiliations:** 1 Gladstone Institutes, San Francisco, California 94158, USA;; 2 Department of Epidemiology and Biostatistics, Institute for Human Genetics, Quantitative Biology Institute, and Institute for Computational Health Sciences, University of California San Francisco, San Francisco, California 94158, USA;; 3 Chan-Zuckerberg Biohub, San Francisco, California 94158, USA

## Abstract

Chromatin interactions and linkage disequilibrium (LD) are both pairwise measurements between genomic loci that show block patterns along mammalian chromosomes. Their values are generally high for sites that are nearby in the linear genome but abruptly drop across block boundaries. One function of chromatin boundaries is to insulate regulatory domains from one another. Since recombination is depressed within genes and between distal regulatory elements and their promoters, we hypothesized that LD and chromatin contact frequency might be correlated genome-wide with the boundaries of LD blocks and chromatin domains frequently coinciding. To comprehensively address this question, we compared chromatin contacts in 22 cell types to LD across billions of pairs of loci in the human genome. These computationally intensive analyses revealed that there is no concordance between LD and chromatin interactions, even at genomic distances below 25 kilobases (kb) where both tend to be high. At genomic distances where LD is approximately zero, chromatin interactions are frequent. While LD is somewhat elevated between distal regulatory elements and their promoters, LD block boundaries are depleted—not enriched—at chromatin boundaries. Finally, gene expression and ontology data suggest that chromatin contacts identify regulatory variants more reliably than do LD and genomic proximity. We conclude that the genomic architectures of genetic and physical interactions are independent, with important implications for gene regulatory evolution, interpretation of genetic association studies, and precision medicine.

Genetic variants ranging from large-scale chromosomal rearrangements to single nucleotide polymorphisms (SNPs) can impact gene function by altering exonic sequence or by changing gene regulation. Recent studies estimate that 93% of disease-associated variants are in noncoding DNA (Welter et al. 2014) and 60% of causal variants map to regulatory elements (Farh et al. 2015), accounting for 79% of phenotypic variance (Gusev et al. 2014). Additionally, disease-associated variants are enriched in regulatory regions (Kundaje et al. 2015), especially those from tissues relevant to the phenotype (Parker et al. 2013). Functionally annotating noncoding variants and correctly mapping causal variants to the genes and pathways they affect is critical for understanding disease mechanisms and using genetics in precision medicine (Kim et al. 2016a; Liang et al. 2017; Lu et al. 2017; Nishizaki and Boyle 2017).

Regulatory variants can affect phenotypes by changing the expression of target genes up to several megabases (Mb) away (Claussnitzer et al. 2015; Kirsten et al. 2015; Javierre et al. 2016; Won et al. 2016), well beyond their LD block (median length ∼1–2 kb) (Supplemental S1B). This prompted Corradin and colleagues to conclude that a gene's regulatory program is not related to local haplotype structure (Corradin et al. 2016). Even when a genome-wide association study (GWAS) SNP is in LD with a gene that has a plausible biological link to the phenotype, the causal variant may be in a nearby noncoding region regulating a different gene (Kim et al. 2016b; Mitchel et al. 2016). Highlighting the long range of regulatory interactions, Mumbach and colleagues found that only 14% of 684 autoimmune variants in T cells targeted their closest gene; 86% skipped one or more intervening genes to reach their target, and 64% of variants interacted with multiple genes (Mumbach et al. 2017). Won and colleagues found that 65% of enhancers in two human brain regions do not interact with their closest gene, and 40% of genes have tissue-specific interactions (Won et al. 2016). Thus, many phenotype-associated variants are in noncoding regions far away and in low LD with the promoters they regulate, and they may be involved in tissue-specific regulatory interactions that genomic distance and LD do not capture.

Distal noncoding variants can cause changes in gene regulation and phenotypes via three-dimensional (3D) chromatin interactions. For example, an obesity-associated FTO variant (rs1421085) was found to disrupt an ARID5B repressor motif in an enhancer for *IRX3/IRX5* during adipocyte differentiation, increasing obesity risk (Claussnitzer et al. 2015). A second study showed that a schizophrenia-associated SNP (rs1191551) regulates the expression of distal gene *FOXG1* in two zones of the developing human cerebral cortex, rather than targeting the nearby gene *PRKD1* (Won et al. 2016). Another example is a SNP associated with papillary thyroid cancer (rs965513) in an LD block containing several enhancer variants that contact the promoter of *FOXE1* and alter its expression (He et al. 2015). In addition, mutagenesis screens identified multiple distal variants that lead to cancer drug resistance by decreasing CUL3 expression (Sanjana et al. 2016). Finally, a common variant linked to five cardiovascular diseases affects the *EDN1* gene via an intermediate common contact site containing a cluster of enhancers (Gupta et al. 2017). These validated causal SNPs demonstrate that regulatory variants can be located far from their target promoters in distinct LD blocks (*IRX3/IRX5* ∼1.2 Mb, *FOXG1* ∼760 kb, *CUL3* ∼100 kb, *FOXE1* ∼60 kb, *EDN1* ∼600 kb), and they can operate via more complex mechanisms than direct SNP-promoter interactions.

New understanding of the 3D genome from high-throughput chromatin capture (Hi-C) and imaging data suggests that regulatory variants and their target genes commonly have low LD. Mammalian genomes are partitioned into regions enriched for chromatin interactions at multiple scales, including topologically associating domains (TADs, median length 880 kb) (Dixon et al. 2012) and contact domains (sub-TADs, median length 250 kb) (Rao et al. 2014). While these chromatin domains resemble the nested block patterns of LD, they have a different origin: insulating chromatin boundary elements that are rarely crossed by chromatin interactions versus frequency of recombination events over generations. Different proteins interact with DNA to mediate these processes: PRDM9 in the case of recombination (Baudat et al. 2010), and structural proteins such as CTCF in the case of chromatin boundaries (Ong and Corces 2014). Thus, one might not expect similarity a priori.

On the other hand, there are several reasons to think that Hi-C and LD maps might be correlated. First, LD is high and chromatin interactions are common at genomic distances <25 kb. Hence, LD and chromatin contact frequencies might be correlated at this scale even though some causal SNPs regulate promoters over long genomic distances where LD is approximately zero. Second, transcribed regions tend to have relatively high LD (McVean et al. 2004; Myers et al. 2005). Extending this finding, Liu and colleagues observed that LD is higher than expected in genomic intervals between distal regulatory elements and their interacting promoters (“recombination valleys”) (Liu et al. 2017). These relationships between regulatory domains and recombination rates raise the possibility of a genome-wide association between chromatin contact frequency and LD due to the strong enrichment of regulatory interactions within versus between chromatin domains (Rao et al. 2014). The relative contributions of these different factors to the chromatin and genetic architectures of the human genome are not known.

To comprehensively evaluate the genome-wide correspondence between interphase chromatin contact maps and LD maps, we conducted a quantitative analysis of billions of pairs of SNPs from the 1000 Genomes Project (The 1000 Genomes Project Consortium 2015) combined with high-resolution Hi-C data from five diverse cell lines (Rao et al. 2014) and promoter capture Hi-C (PCHi-C) data from 17 primary blood cell types (Javierre et al. 2016). To link our findings to functional regulatory variation in a consistent cellular context, we integrated the blood cell chromatin interaction data with B cell expression quantitative trait loci (eQTL) (Fairfax et al. 2012) and blood-relevant phenotypes from the GWAS catalog (MacArthur et al. 2017). This massive analysis showed that LD and Hi-C maps are uncorrelated.

## Results

To comprehensively compare the genomic architectures of LD and chromatin contacts, we generated two types of data structures from publicly available data (Supplemental Table S1). The first includes LD blocks and pairwise LD between all high-quality, bi-allelic SNPs across individuals from each of the 1000 Genomes Project super-populations (AFR: African, AMR: Admixed American, EAS: East Asian, EUR: European, SAS: South Asian) (The 1000 Genomes Project Consortium 2015). The second records contact frequencies between all pairs of fragments in 22 human cell types with high-resolution Hi-C (Rao et al. 2014) or promoter capture Hi-C (Javierre et al. 2016) data that measure interactions between baited promoters and promoter interacting regions (PIRs). These chromatin contact data were used to generate lists of statistically significant interacting regions and distance-matched regions with nonsignificant interactions for each cell type using methods that account for expected contact frequencies and adjust for multiple testing. Significant chromatin interactions from Hi-C were computed using Juicer (Durand et al. 2016) and represent statistical enrichment of contacts over a particular choice of local background, whereas those from PCHi-C were computed using CHiCAGO (Cairns et al. 2016) and indicate if a region is likely to be in the same contact domain with a promoter or not. Due to the resolution of chromatin interaction assays, we could not compare LD to 3D proximity of sites separated by <5 kb where both values are expected to be high.

We implemented efficient algorithms to perform computationally intensive analyses spanning these approximately 1.6 million LD blocks, 27 billion SNP pairs, and 3.1 million statistically significant chromatin interactions (Supplemental Table S1). By analyzing the genome-wide relationship between LD and chromatin contacts from multiple perspectives, we show that LD is not correlated with chromatin interaction frequency. Our results also demonstrate that chromatin interactions are better than LD and genomic distance at capturing functional relationships between noncoding SNPs and the genes and phenotypes they regulate.

### Chromatin interactions and LD have different genomic architectures

**Figure 1.**
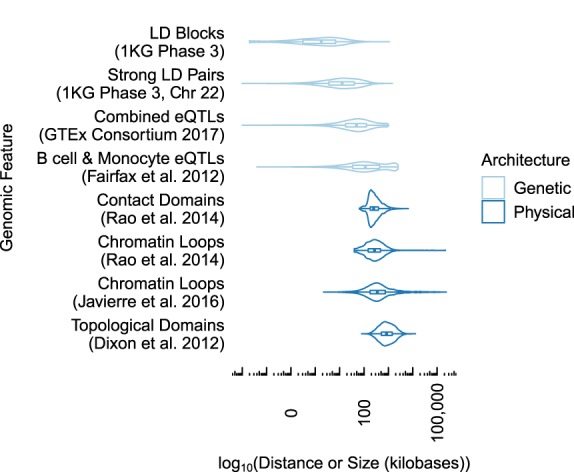
LD blocks and strong LD pairs (*R*^2^ > 0.8) operate across tens of kb or less, while chromatin interactions and multiscale domains enriched for chromatin interactions span hundreds of kb. eQTLs lie roughly in between. Summaries are computed over all super-populations, tissue types, or cell types in each data set.

**Figure 2.**
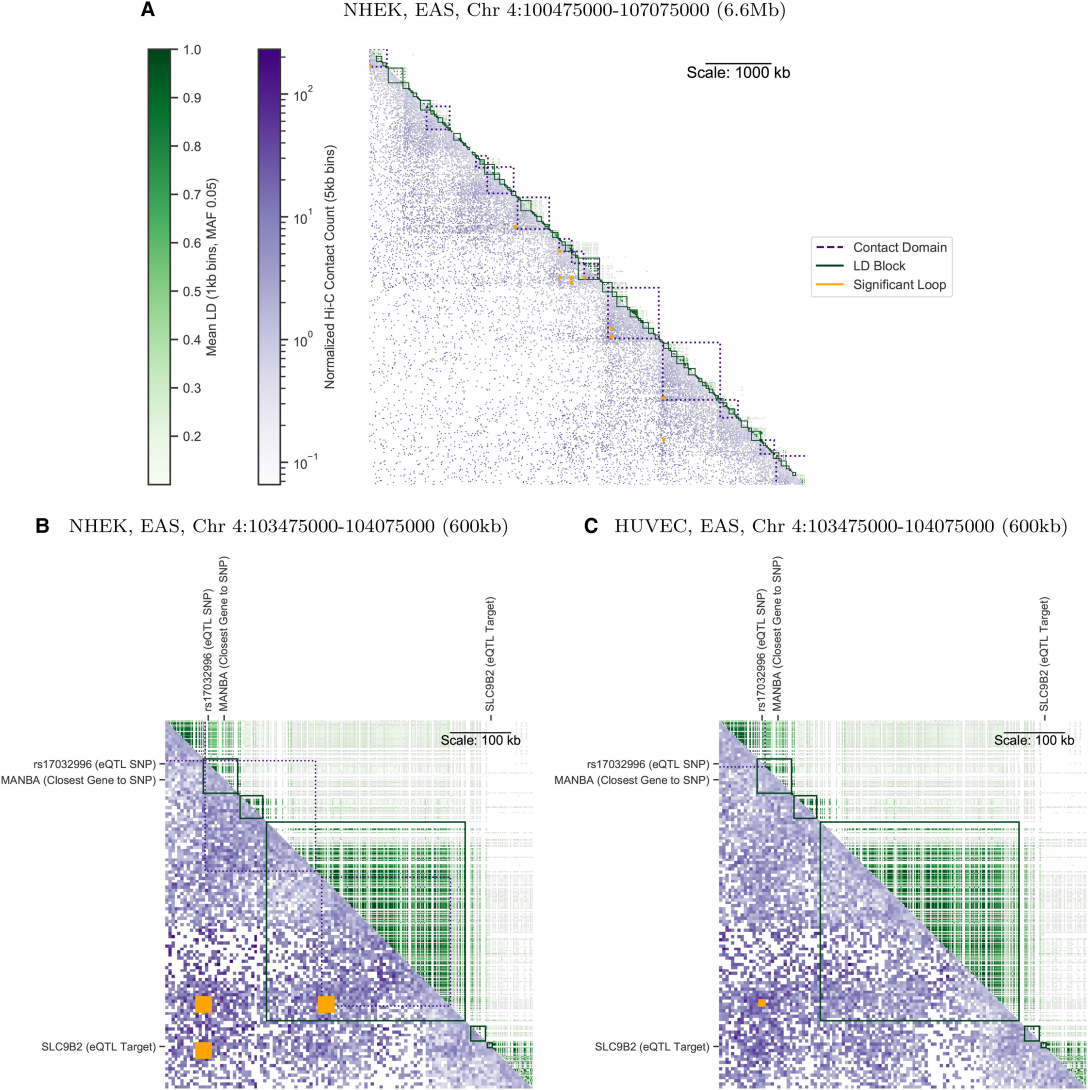
An annotated matrix illustrates differences between the genomic scales of LD (The 1000 Genomes Project Consortium 2015) (*R*^2^, *upper* triangle, green) versus Hi-C contact frequency (Rao et al. 2014) (*lower* triangle, purple). Rows and columns are binned genomic coordinates (hg19 assembly) with *lower* bins near the *upper left*; for example, row 10 column 11 stores the LD between a bin and its neighbor, while row 11 column 10 stores the contact frequency for the same pair of bins. More frequent contacts (5-kb bins) are darker purple; higher LD (averaged over nonzero LD pairs in 1-kb bins) are darker green. Contact domains (nested purple squares) and significant interactions (orange squares) were computed from Hi-C data. LD blocks (green squares) were computed from 1000 Genomes genotypes. While some LD blocks fall within contact domains, there are also many cases where they overlap domain boundaries. (*A*) A representative 6.6-Mb locus on Chromosome 4 shows Hi-C contacts (NHEK cells) span much longer distances than LD (EAS super-population). (*B*) A 600-kb locus on the same chromosome illustrates the complexities of mapping a noncoding SNP (rs17032996) to a target gene. The closest gene *MANBA* falls within the same LD block as the SNP. However, Hi-C data shows the SNP contacts the *SLC9B2* gene ∼460 kb away in NHEK cells, skipping over intervening expressed gene *MANBA*. rs17032996 is also an eQTL in B cells (Fairfax et al. 2012) and significantly interacts with *SLC9B2* in several blood cell types (Javierre et al. 2016). (*C*) In HUVEC cells, the SNP no longer interacts with *SLC9B2*, and several contact domains are lost.

Both LD and chromatin contact frequency measure the strength of a relationship between pairs of genomic sites. However, these two measures differ fundamentally in their scales: Chromatin contacts span much longer distances (Fig. 1). Genetic architecture forms LD blocks of median length 2 kb (combined 1000 Genomes super-populations) in which a percentage of SNP pairs exceed a common threshold of *R*^2^ > 0:8 (Gabriel et al. 2002). Most SNPs in the genome are located in LD blocks several kb or less (Supplemental Fig. S1), though strong LD pairs have a median distance of 13 kb, as they can be located in different blocks. On the other hand, the 3D architecture of chromatin forms regions enriched for interactions at much longer scales, including focal interactions (median 270 kb) (Rao et al. 2014) (median 350 kb) (Javierre et al. 2016), contact domains (median 250 kb) (Rao et al. 2014), and TADs (median 840 kb) (Dixon et al. 2012). This difference is evident when contact frequency from a particular cell type is plotted alongside LD from any of the 1000 Genomes super-populations, both at the scale of TADs (Fig. 2A) and within smaller contact domains (Fig. 2B) where chromatin interactions are frequent but LD structure is low or limited to smaller LD blocks. Due to this difference in scale, noncoding SNPs frequently contact genes located hundreds of kb away without being in LD with those genes (Fig. 2B). These distal chromatin interactions may differ across cell types, whereas LD does not (Fig. 2C). A similar example is shown in Supplemental Figure S2.

**Figure 3.**
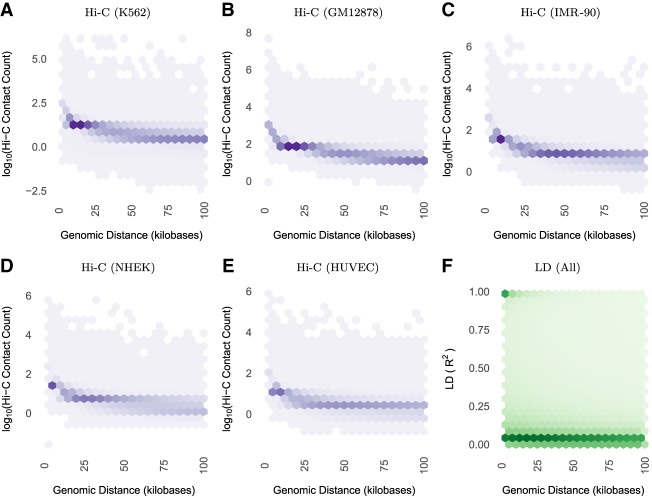
Both (*A*–*E*) Hi-C contact frequency (Rao et al. 2014) and (*F*) LD are anti-correlated with genomic distance (Spearman's correlation between −0.50 and −0.71 for Hi-C across cell lines; it is −0.52 for LD). LD decays toward zero at much shorter genomic distances than contact frequency, with high LD SNP pairs concentrated below 50 kb. In contrast, Hi-C contacts are common up to and exceeding the median length of contact domains (250 kb) or TADs (840 kb). All plots display nonzero values from their respective data sets. Contact frequencies (panels *A*–*E*) vary in approximate proportion to sequencing depth and number of replicates per cell line (Supplemental Table S1). Panel *F* includes 836 million bi-allelic SNP pairs on Chromosome 14, which is representative of other chromosomes. Supplemental Figure S3 shows decay up to 2 Mb, while this figure highlights decay up to 100 kb. Supplemental Figure S4 shows that there is nearly identical LD scaling per super-population.

To quantify the decay rates of LD versus chromatin contacts genome-wide, we analyzed all pairs of sites separated by a given genomic distance with respect to Hi-C contact frequency (Rao et al. 2014) versus LD in 1000 Genomes individuals. This showed that contact frequency decays with genomic distance much slower than LD both across (Fig. 3; Supplemental Fig. S3) and within human populations (Supplemental Figs. S4, S5). Furthermore, statistically significant chromatin interactions occur between genomic regions separated by dozens, hundreds, or even thousands of LD blocks (Supplemental Fig. S6A,B), while most SNP pairs with nonzero LD cross 0-2 contact domains (Supplemental Fig. S6C). PCHi-C data shows the same broad trends (Supplemental Fig. S7). In summary, genetic and physical architectures of human chromosomes differ at multiple scales.

### Chromatin contact frequencies have low concordance with LD across genomic distances

**Figure 4.**
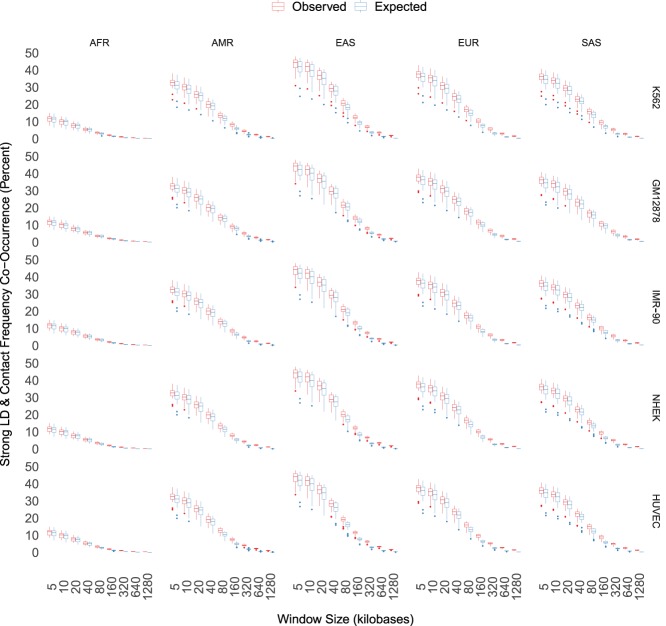
Frequent Hi-C contacts (top 25%) and strong LD (*R*^2^ > 0.8) co-occur <50% of the time at short genomic distances. Concordance is cut nearly in half at 40 kb, where most LD has decayed to 0, and is nearly 0 at many scales where statistically significant chromatin interactions occur. Maximum concordance and rate of decay vary by super-population, with AFR having only ∼12% concordance at short genomic distances. LD is elevated compared to expectation at the longest genomic distances, although the effect size is small and median LD is close to zero.

Contact frequency and LD could still be correlated at shorter genomic distances where LD is more often nonzero. To explore this possibility, we analyzed the concordance of frequent Hi-C contacts (top 25% of contact frequencies) and strong LD values (*R*^2^ > 0:8) for pairs of sites separated by distances ranging from 5 kb to 1.2 Mb (Methods). As expected, chromatin interactions and strong LD co-occur most often for pairs of sites <10 kb apart, ranging across super-populations from ∼10% (AFR) to 40% (EAS) of site pairs, while being fairly consistent across cell types within a super-population. This level of concordance is similar to what is expected if there is no association between the two variables (Fig. 4), and it decays rapidly with genomic distance as significant chromatin interactions continue to occur but average LD approaches zero in each super-population (Fig. 3). Frequent Hi-C contacts and strong LD do co-occur significantly more than expected at genomic distances beyond 100 kb, consistent with recombination valleys between regulatory elements and target genes at this genomic distance (Liu et al. 2017). However, this effect is very small in magnitude, and both the observed and the expected rate are close to zero. In other words, frequent Hi-C contacts over 100 kb have higher LD than distance-matched noninteracting sites, but most Hi-C contacts are not in LD. Together, these patterns suggest that concordance between frequent chromatin interactions and LD is largely driven by the genomic architecture of LD.

### LD is not elevated in significant chromatin interactions

**Figure 5.**
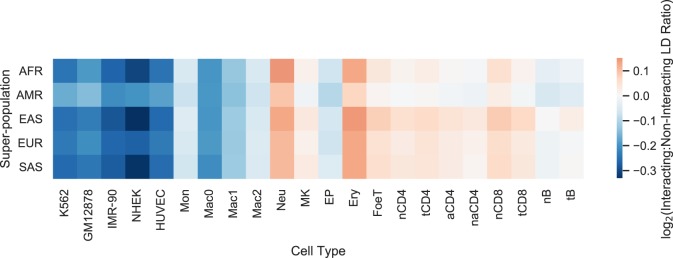
The maximum pairwise LD between SNPs located on the fragments of statistically significant and distance-matched nonsignificant chromatin interactions (interaction LD) was computed for five Hi-C and 17 PCHi-C data sets. The log ratio of mean interaction LD for significant versus nonsignificant interactions quantifies how well LD acts as a proxy for chromatin interactions; a log ratio greater than 0 indicates significant interactions are enriched for SNPs in strong LD. However, the log ratio is near 0 for all cell types and super-populations, indicating that LD is not a sufficient proxy for chromatin interactions. Supplemental Table S2 provides numeric values for this figure; log ratios smaller or larger than 0 are the result of relatively small differences in weak LD.

Next, we compared LD and chromatin structure focusing on statistically significant chromatin interactions, as these might harbor high LD SNPs even if less frequent chromatin contacts are rarely genetically linked. For each statistically significant and distance-matched nonsignificant interaction, we computed the maximum LD between pairs of SNPs occurring on opposing fragments. The log ratio of interacting versus noninteracting fragment LD is close to 0 across all super-populations and cell types (Fig. 5; Supplemental Table S2), indicating no elevation of LD at interacting regions. In addition, LD is very low between noncoding regions and interacting promoters in PCHi-C data, with 2%–7% of interacting fragments located within the same LD block (Table 1). Thus, the overall trend of low concordance between chromatin interaction frequency and LD is also observed at the most frequently interacting regions of the human genome.

**Table 1.**
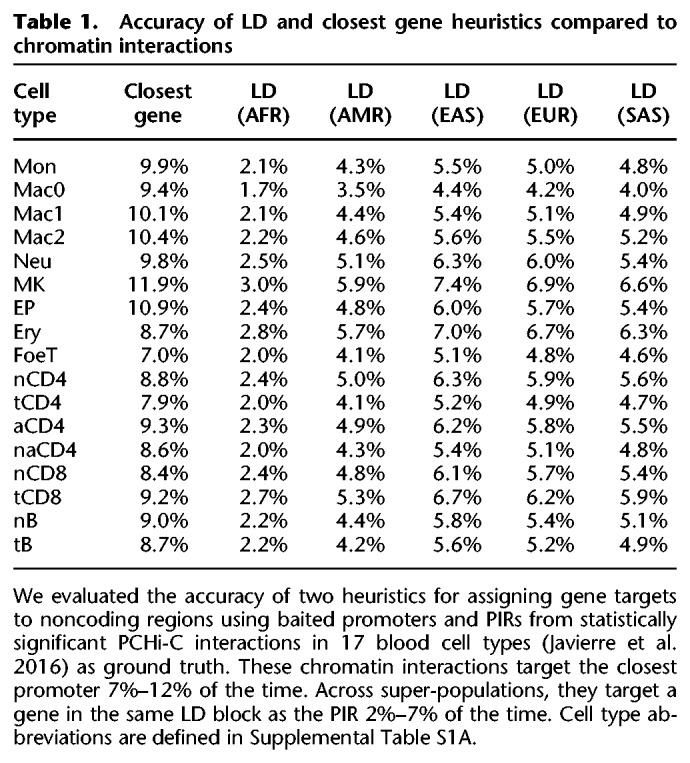
Accuracy of LD and closest gene heuristics compared to chromatin interactions

### LD is elevated across chromatin boundaries

In our final comparison of LD and chromatin interaction maps, we examined patterns of LD around chromatin domain boundaries. For each super-population, we evaluated the distribution of LD block sizes as a function of distance to the nearest GM12878 contact domain boundary. We found that contact domain boundaries tend to be spanned by some of the longest LD blocks in the human genome (Supplemental Fig. S8A,B). To further explore this pattern, we compared the median distance between GM12878 contact domain boundaries and their nearest LD block boundary to the distribution of median distances after permuting LD block locations. For all super-populations, the observed median distance is significantly longer than expected (Supplemental Fig. S8C). These results show that LD block boundaries do not coincide with chromatin domain boundaries, and LD is, in fact, elevated across chromatin boundaries.

### LD is low between distal regulatory SNPs and their genes

**Figure 6.**
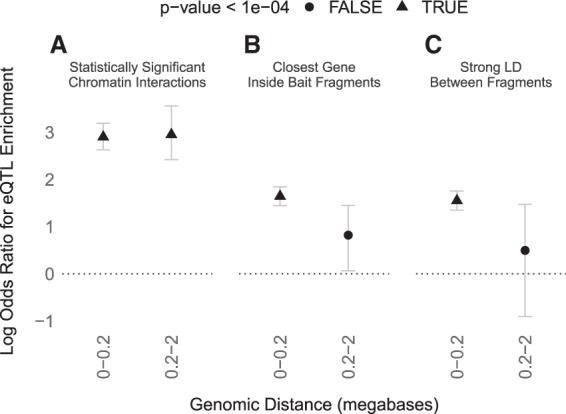
(*A*) B cell eQTLs (Fairfax et al. 2012) are significantly enriched in statistically significant B cell PCHi-C interactions (Javierre et al. 2016) at both proximal (<200 kb) and distal (200 kb to 2 Mb) distances from their promoters, with an odds ratio of ∼20 (natural log odds ratio of ∼3). (*B*) eQTLs are significantly enriched only at proximal distances and with smaller odds ratios (∼4) when conditioning on bait fragments containing the closest gene to the promoter-interacting fragment, or (*C*) conditioning on interacting fragments being in strong LD (maximum pairwise *R*^2^ > 0.8 for any super-population) with bait fragments. Whiskers are 95% confidence intervals.

Genetic variants associated with statistically significant differences in a gene's expression (eQTLs) provide evidence of functional relationships between regulatory regions and genes separated by long genomic distances. Indeed, target genes of GTEx eQTLs (GTEx Consortium 2017) and blood eQTLs (Fairfax et al. 2012) have median distances of 49 and 113 kb, respectively. Combined with our other findings, these distances suggest that a distal eQTL and its target gene are likely to have zero LD and thus be separated by a large number of LD blocks (though this is not universally true). We therefore compared the frequency of eQTLs among noncoding regions that interact with gene promoters versus distance-matched regions that do not, using B cells where both PCHi-C (Javierre et al. 2016) and eQTL (Fairfax et al. 2012) data are available. Across super-populations, we found that statistically significant chromatin interactions are highly enriched for eQTLs across genomic distances up to 2 Mb (Figs. 1, 6; Supplemental Fig. S9), consistent with previous studies (Kirsten et al. 2015). In contrast, regions in strong LD with a promoter are only enriched for eQTLs at genomic distances <200 kb, and the odds ratios for these proximal eQTLs are smaller (∼4 versus ∼20). Thus, both distal and proximal eQTLs are more accurately mapped to their promoters with PCHi-C data than with genomic distance or LD. These results emphasize that eQTLs are often in 3D proximity to their target promoters regardless of genomic distance, despite having low or zero LD.

### Mapping noncoding variants to genes with Hi-C produces more functional enrichments than genomic distance or LD

**Figure 7.**
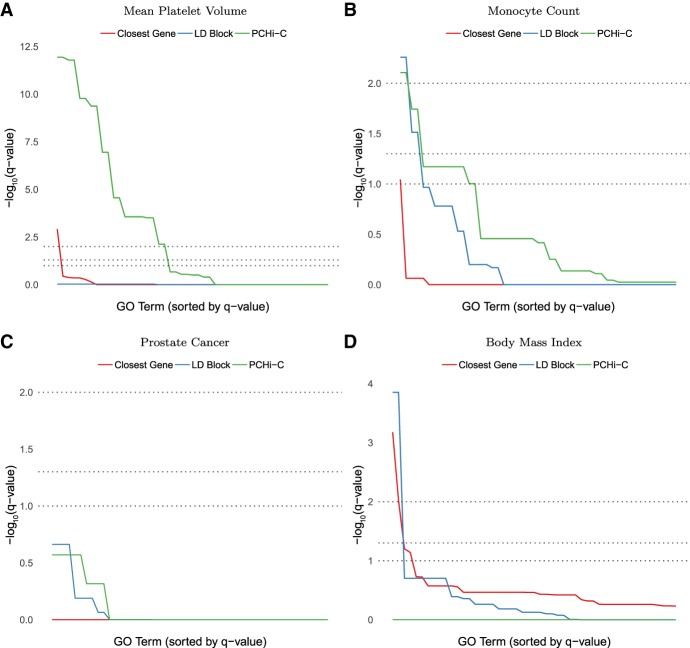
Enrichment of GO terms (Benjamini-Hochberg–adjusted *P*-values, −log_10_ scale) in multiple phenotypes from the GWAS catalog (MacArthur et al. 2017). Methods for functional assignment of SNPs include using the closest gene, all genes within the same LD block (EUR super-population), and promoter capture bait genes with a SNP located in the promoter-interacting region of a statistically significant blood cell chromatin interaction (Javierre et al. 2016). Gray horizontal lines indicate false discovery rate (FDR) cutoffs of 1%, 5%, and 10%. (*A*,*B*) In blood-relevant phenotypes, PCHi-C bait genes interacting in 3D with GWAS SNPs typically show more enrichment for a larger number of terms than same-LD-block or closest gene approaches, whose enrichment is affected by large numbers of false positives and negatives. (*C*,*D*) For non-blood phenotypes, chromatin interactions in the wrong cell type can have little or no enrichment. Statistically significant enrichments are occasionally detected with the closest gene and same-LD-block approaches, perhaps reflecting the cell-type specificity of PCHi-C over these approaches.

If regulatory interactions are common at large genomic distances where LD is approximately zero, then GWAS SNPs linked to genes via Hi-C should include more true gene targets than using closest genes or genes in LD with the SNP (even though noncoding SNPs can tag causal coding SNPs). If true, then the set of genes associated with GWAS hits via Hi-C should also share more functional annotations. To test this idea, we examined the magnitude and statistical significance of Gene Ontology (GO) enrichments for genes associated with all GWAS SNPs for a given phenotype via PCHi-C interactions (all blood cell types, all genes with PIRs overlapping the SNP) (Javierre et al. 2016), genomic distance (closest promoter to the SNP), or genetic distance (all promoters in the same LD block as the SNP). These statistical tests account for differences in the numbers of genes associated with SNPs by each method and are robust to correlations between GO terms (Methods). Most blood-relevant phenotypes in the GWAS catalog (MacArthur et al. 2017) had the largest number of significantly enriched GO terms using blood cell PCHi-C assignment (Fig. 7A,B) compared to LD or closest gene approaches. LD-based assignment occasionally produced a limited number of terms with more significant adjusted *P*-values than those from PCHi-C assignment (Fig. 7B). Nonetheless, using LD resulted in fewer GO terms associated with the phenotype and a lower area-under-the-curve than PCHi-C. As a negative control, we examined GWAS SNPs for phenotypes not relevant to blood and found few significant GO terms (Fig. 7C), as expected. With these negative control phenotypes, the closest gene and LD approaches sometimes have a number of significant GO enrichments while PCHi-C does not (Fig. 7D), confirming that PCHi-C enrichments are tissue-specific. These results highlight the need for chromatin interaction data collected in the cell type of interest to avoid false positive GO enrichments and to harness the power of chromatin structure for functional assignment.

## Discussion

Chromatin interactions and LD are both pairwise measurements between genomic loci that show block patterns along mammalian chromosomes. Given their similar structure, it might be tempting to speculate that LD blocks correspond to or are contained within three-dimensional chromatin domains. On the other hand, there is growing awareness that regulatory interactions need not be in LD or nearby on the genome, with many recent examples of distal enhancers and eQTLs documented in the literature. We also know that chromatin domains and LD blocks have different origins (PRDM9-mediated recombination versus CTCF-mediated insulation of regulatory regions). Despite these compelling hypotheses and examples, the correlation of LD and chromatin interaction maps has not been quantified systematically genome-wide until now. To address this question, we developed diverse, computationally efficient statistical analyses to compare genome-wide LD and chromatin interaction maps across different length scales. Leveraging five cell lines (Rao et al. 2014), 17 human primary blood cell types (Javierre et al. 2016), and five super-populations from the 1000 Genomes Project (The 1000 Genomes Project Consortium 2015), we discovered that human LD maps are not correlated with chromatin interaction maps at genomic distances of 5 kb or more.

The main factor driving these differences is the frequency of chromatin interactions over genomic distances where the genetic linkage between SNPs is close to zero. Significant chromatin interactions often span hundreds or sometimes thousands of LD blocks. This result does not contradict the existence of recombination valleys between distal regulatory regions and their promoters at ∼100–200 kb (Liu et al. 2017). This is because (1) regulatory interactions are a subset of all distal chromatin interactions, and (2) despite higher than expected LD within regulatory domains, most distal regulatory SNPs and their target promoters have zero LD. Even at genomic distances where contact frequencies and LD are both high on average, the correlation between their block patterns is weak. Furthermore, some of the largest LD blocks in the human genome span chromatin domain boundaries. This suggests that chromatin boundaries may be recombination cold spots for some of the same reasons as regulatory domains (e.g., methylation or closed chromatin in the germ line) (Liu et al. 2017). Together, these results clarify on a genome-wide scale that human recombination patterns and interphase chromatin organization are largely uncorrelated.

This study has important implications for associating noncoding variants with genes, downstream phenotypes, and molecular mechanisms. Our results verify on a genome-wide scale that variants have great potential to regulate genes beyond their LD block. This holds consistently across super-populations and cell types. Hence, mapping candidate regulatory variants to the closest gene or other genes in the same LD block will typically miss most chromatin interactions between the variant and gene promoters. In addition, LD is the same in all cell types whereas within-TAD regulatory interactions vary across cell types (Fig. 2; Supplemental Fig. S2; Rao et al. 2014). However, genomic distance is easy to compute and thus continues to be used despite its known shortcomings. In contrast, chromatin interaction data of sufficient resolution (∼1–5 kb) for linking specific regulatory variants to promoters is available in limited cell types and can be expensive to generate. This has slowed the adoption of chromatin interaction data as a new paradigm for mapping noncoding variants (e.g., GWAS loci) to genes. Our findings underscore the importance of generating or computationally predicting chromatin structure across many more cell types.

In addition to highlighting the need for incorporating chromatin interactions into functional assignment, the discordance between chromatin contact frequency and LD has evolutionary implications. One consequence is that entire TADs or sub-TADs do not typically segregate as single haplotypes in human populations, enabling independent selection on regulatory variants versus the promoter and coding variants of their target genes. Furthermore, large LD blocks that span chromatin domain boundaries indicate that regulatory and coding variants from one domain can segregate with variants from the adjacent domain. The fact that haplotype breakpoints do not align with chromatin boundaries may indicate that recombination is deleterious at these functional elements. These findings are different from observations regarding fixed structural differences between genomes of various mammals, which tend to preserve TADs with breakpoints enriched at TAD boundaries (Krefting et al. 2018; Lazar et al. 2018). We therefore conclude that, while chromatin domains are functional genomic entities maintained as syntenic units over evolutionary time, recombination is largely independent of interphase chromatin structure. This creates novel haplotypes of the genomic elements within TADs on which selection can operate.

## Methods

In order to perform large scale analyses, some caveats were necessary in order to place reasonable bounds on compute time and memory, even in a high-performance computing environment. For example, LD was computed between SNP pairs up to 2 Mb apart and stored if LD was 0.01 or greater. Also, the resolution of Hi-C and PCHi-C data prevented examining correlations between chromatin interactions and LD at genomic distances below 5 kb.

Hi-C data (Rao et al. 2014) was obtained from the NCBI Gene Expression Omnibus using accession GSE63525, including contact domains, statistically significant interactions, and sparse contact matrices along with coefficients for normalization and expectation. Promoter capture Hi-C data (Javierre et al. 2016) was obtained from Open Science Framework (https://osf.io/u8tzp). These data sets were aligned to hg19 by their respective authors and were not realigned to hg38 to facilitate direct comparison with their results. Given the observed levels of statistical significance, we do not expect these results to depend on the use of hg19.

Analyses utilized BCFtools 1.6 (Li 2011), BEDTools 2.27.1 (Quinlan and Hall 2010), PLINK 1.90b5 (Chang et al. 2015), pandas 0.22.0 (McKinney 2012), Matplotlib 2.1.1 (Hunter 2007), seaborn 0.8.1 (http://doi.org/10.5281/zenodo.883859), ggplot 2.2.1 (Wickham 2009), and GNU Parallel 20171222 (Tange 2011). Python 3.6.4 was provided by the Miniconda distribution; R 3.4.3 (R Core Team 2018) was compiled from source using gcc 7.2.1.

### Linkage disequilibrium

Bi-allelic SNPs from phase 3 of the 1000 Genome Project were first converted to PLINK's binary BED format (–make-bed –allow- extra-chr –biallelic-only), and the pairwise LD computed (–r2 dprime) for all SNPs with a minimum MAF of 5% (–maf 0.05) located within 2 Mb of each other (–ld-window-kb 2000). The number of pairwise comparisons allowed within a window was increased (–ld-window 10000), and the default R^2^ filter lowered from 20% down to 1% (–ld-window-r2 0.01). Pairs below this threshold were assigned *R*^2^ = 0. LD computations were performed separately for each super-population by using the 1000 Genomes panel file (integrated call samples v3.20130502.ALL.panel) and the –filter flag.

LD blocks were computed using PLINK (utilizing the algorithm from Gabriel et al. 2002) with the –blocks no-pheno-req no-small-max-span –blocks-max-kb 2000 flags. Blocks were computed separately for each super-population using the same –filter flag and panel file.

### Interacting versus noninteracting LD

For each 1000 Genomes super-population, bi-allelic SNPs with a minimum MAF of 5% were intersected with either Hi-C (Rao et al. 2014) or promoter capture Hi-C (Javierre et al. 2016) fragments using the bedtools pairtobed command with the -type both flag. For each pair of interacting fragments, the maximum LD between SNPs on different fragments was computed. The mean of this maximum pairwise LD was computed separately for statistically significant and nonsignificant interactions in order to compute a ratio.

Raw chromatin interaction data was processed by tools that assess the statistical significance of interactions between pairs of loci. Hi-C data was processed by Juicer (Durand et al. 2016), while PCHi-C was processed by CHiCAGO (Cairns et al. 2016). These tools have several differences (detailed in their respective publications) including whether loci are first binned to improve statistical power, as well as models for the null distribution of chromatin contacts. Statistically significant Hi-C interactions (positives) were generated from binned contact matrices using the Juicer pipeline at 10% false discovery rate (FDR) (GEO accession GSE63525). Binned interactions were shuffled along the same chromosome (bedtools shuffle -chrom) to obtain distance-matched nonsignificant interactions (negatives). PCHi-C positives and negatives were obtained from a list of interactions scored by the CHiCAGO pipeline (https://osf.io/u8tzp). Following the original paper, interactions with a score less than five were treated as negatives and were distance-matched to positives using quantile binning of interaction distance.

### Hi-C versus LD concordance

Observed over expected Hi-C values were computed using formulas from Rao et al. (2014) applied to VC-normalized contact counts at 5-kb resolution for each cell line. For comparable resolution, LD per 5-kb genomic bin was computed for each 1000 Genomes super-population using the 75th percentile of pairwise LD values in the bin. This was more robust to outliers and heavily zero-skewed LD distributions than the average or median.

Concordance was computed based on whether a bin's LD value was strong (*R*^2^ > 0:8) and its chromatin contact frequency was strong (above the 75th percentile of contact frequencies) for all bins located in nonoverlapping genomic windows of fixed size. This was repeated for window sizes of 5, 10, 20, 40, 80, 160, 320, 640, and 1280 kb to examine concordance across multiple scales and without variation introduced by different TAD-calling algorithms.

### eQTL statistics

B cell eQTL coordinates (Fairfax et al. 2012) were intersected with naive B cell promoter capture Hi-C interactions (Javierre et al. 2016); the eQTL was required to overlap the promoter-interacting region and the eQTL target was required to overlap the bait fragment. The presence or absence of an interacting eQTL was stored in a binary vector. Next, the closest gene to each promoter-interacting fragment was computed using BEDTools closest and Ensembl gene annotations. The presence or absence of the closest gene in the corresponding bait fragment was stored in a binary vector. Next, the statistical significance of chromatin interactions (thresholded using a CHiCAGO score of 5) was stored in a binary vector. Finally, a vector stored the maximum pairwise LD between fragments for each super-population.

eQTLs were tested for enrichment in (1) statistically significant chromatin interactions, (2) interactions where the bait was the closest gene, and (3) interactions where the maximum pairwise LD between fragments was greater than 0.8 for any super-population. Interactions were quantile binned by distance up to 2 Mb to prevent zero-count entries in the contingency table. Odds ratios, *P*-values, and confidence intervals were computed using logistic regression (R's glm function with family = ‘binomial’ and the confint function) for each distance bin.

### Gene Ontology enrichment

The promoter-interacting region of statistically significant PCHi-C interactions (Javierre et al. 2016) was intersected with SNPs for the 30 most abundant phenotypes in the GWAS catalog (release 2018-01-31) (MacArthur et al. 2017). For each phenotype and each GO term, a Fisher's exact test was computed on a 2 by 2 contingency table counting if the interaction contained a GWAS SNP for the phenotype in its PIR and whether or not the interaction's bait gene was annotated with that GO term. Fisher's exact test is significant if more genes interact with a GWAS SNP and have the GO term than expected given the counts of genes with and without GWAS SNPs as well as the counts of genes with and without the GO term (i.e., conditional on the marginal totals in the 2 by 2 table). This makes the resulting *P*-value conditional on gene counts, facilitating comparisons across phenotypes and GO terms. Fisher's exact test is also possible and has reasonable power when gene counts are low. To correct for multiple hypothesis testing, we applied the Benjamini-Hochberg false discovery rate adjustment to the resulting *P*-values. For comparison, this was repeated for the closest gene to each GWAS SNP, as well as all genes in the same LD block as the GWAS SNP. We note that the hierarchical structure of GO will result in correlations between tests for related GO terms. The Benjamini-Hochberg adjustment is robust to this type of clustered dependence (positive regression dependence). While the number of significant GO terms is expected to be higher in parts of the GO hierarchy with more nested terms, this inflation should affect all three ways of mapping GWAS SNPs to genes equally.

### Software availability

Source code is available as Supplemental File S1 and from GitHub (https://github.com/shwhalen/loopdis).

## Supplementary Material

Supplemental Material

## References

[GR238022WHAC1] The 1000 Genomes Project Consortium. 2015 A global reference for human genetic variation. Nature 526: 68–74. 10.1038/nature15393 26432245 PMC4750478

[GR238022WHAC2] Baudat F, Buard J, Grey C, Fledel-Alon A, Ober C, Przeworski M, Coop G, de Massy B. 2010 PRDM9 is a major determinant of meiotic recombination hotspots in humans and mice. Science 327: 836–840. 10.1126/science.1183439 20044539 PMC4295902

[GR238022WHAC3] Cairns J, Freire-Pritchett P, Wingett SW, Várnai C, Dimond A, Plagnol V, Zerbino D, Schoenfelder S, Javierre BM, Osborne C, 2016 CHiCAGO: robust detection of DNA looping interactions in Capture Hi-C data. Genome Biol 17: 127 10.1186/s13059-016-0992-2 27306882 PMC4908757

[GR238022WHAC4] Chang CC, Chow CC, Tellier LC, Vattikuti S, Purcell SM, Lee JJ. 2015 Second-generation PLINK: rising to the challenge of larger and richer datasets. GigaScience 4: 7 10.1186/s13742-015-0047-8 25722852 PMC4342193

[GR238022WHAC5] Claussnitzer M, Dankel SN, Kim KH, Quon G, Meuleman W, Haugen C, Glunk V, Sousa IS, Beaudry JL, Puviindran V, 2015 *FTO* obesity variant circuitry and adipocyte browning in humans. N Engl J Med 373: 895–907. 10.1056/NEJMoa1502214 26287746 PMC4959911

[GR238022WHAC6] Corradin O, Cohen AJ, Luppino JM, Bayles IM, Schumacher FR, Scacheri PC. 2016 Modeling disease risk through analysis of physical interactions between genetic variants within chromatin regulatory circuitry. Nat Genet 48: 1313–1320. 10.1038/ng.3674 27643537 PMC5083135

[GR238022WHAC7] Dixon JR, Selvaraj S, Yue F, Kim A, Li Y, Shen Y, Hu M, Liu JS, Ren B. 2012 Topological domains in mammalian genomes identified by analysis of chromatin interactions. Nature 485: 376–380. 10.1038/nature11082 22495300 PMC3356448

[GR238022WHAC8] Durand NC, Shamim MS, Machol I, Rao SS, Huntley MH, Lander ES, Aiden EL. 2016 Juicer provides a one-click system for analyzing loop-resolution Hi-C experiments. Cell Syst 3: 95–98. 10.1016/j.cels.2016.07.002 27467249 PMC5846465

[GR238022WHAC9] Fairfax BP, Makino S, Radhakrishnan J, Plant K, Leslie S, Dilthey A, Ellis P, Langford C, Vannberg FO, Knight JC. 2012 Genetics of gene expression in primary immune cells identifies cell type–specific master regulators and roles of HLA alleles. Nat Genet 44: 502–510. 10.1038/ng.2205 22446964 PMC3437404

[GR238022WHAC10] Farh KKH, Marson A, Zhu J, Kleinewietfeld M, Housley WJ, Beik S, Shoresh N, Whitton H, Ryan RJH, Shishkin AA, 2015 Genetic and epigenetic fine mapping of causal autoimmune disease variants. Nature 518: 337–343. 10.1038/nature13835 25363779 PMC4336207

[GR238022WHAC11] Gabriel SB, Schaffner SF, Nguyen H, Moore JM, Roy J, Blumenstiel B, Higgins J, DeFelice M, Lochner A, Faggart M, 2002 The structure of haplotype blocks in the human genome. Science 296: 2225–2229. 10.1126/science.1069424 12029063

[GR238022WHAC12] GTEx Consortium. 2017 Genetic effects on gene expression across human tissues. Nature 550: 204–213. 10.1038/nature24277 29022597 PMC5776756

[GR238022WHAC13] Gupta RM, Hadaya J, Trehan A, Zekavat SM, Roselli C, Klarin D, Emdin CA, Hilvering CR, Bianchi V, Mueller C, 2017 A genetic variant associated with five vascular diseases is a distal regulator of endothelin-1 gene expression. Cell 170: 522–533.e15. 10.1016/j.cell.2017.06.049 28753427 PMC5785707

[GR238022WHAC14] Gusev A, Lee SH, Trynka G, Finucane H, Vilhjalmsson BJ, Xu H, Zang C, Ripke S, Bulik-Sullivan B, Stahl EA, 2014 Partitioning heritability of regulatory and cell-type-specific variants across 11 common diseases. Am J Hum Genet 95: 535–552. 10.1016/j.ajhg.2014.10.004 25439723 PMC4225595

[GR238022WHAC15] He H, Li W, Liyanarachchi S, Srinivas M, Wang Y, Akagi K, Wang Y, Wu D, Wang Q, Jin V, 2015 Multiple functional variants in long-range enhancer elements contribute to the risk of SNP rs965513 in thyroid cancer. Proc Natl Acad Sci 112: 6128–6133. 10.1073/pnas.1506255112 25918370 PMC4434723

[GR238022WHAC16] Hunter JD. 2007 Matplotlib: a 2D graphics environment. Comput Sci Eng 9: 90–95. 10.1109/MCSE.2007.55

[GR238022WHAC17] Javierre BM, Burren OS, Wilder SP, Kreuzhuber R, Hill SM, Sewitz S, Cairns J, Wingett SW, Varnai C, Thiecke MJ, 2016 Lineage-specific genome architecture links enhancers and non-coding disease variants to target gene promoters. Cell 167: 1369–1384.e19. 10.1016/j.cell.2016.09.037 27863249 PMC5123897

[GR238022WHAC18] Kim K, Jang K, Yang W, Choi EY, Park SM, Bae M, Kim YJ, Choi JK. 2016a Chromatin structure-based prediction of recurrent noncoding mutations in cancer. Nat Genet 48: 1321–1326. 10.1038/ng.3682 27723759

[GR238022WHAC19] Kim MJ, Yu CY, Theusch E, Naidoo D, Stevens K, Kuang YL, Schuetz E, Chaudhry AS, Medina MW. 2016b SUGP1 is a novel regulator of cholesterol metabolism. Hum Mol Genet 25: 3106–3116. 10.1093/hmg/ddw151 27206982 PMC5181593

[GR238022WHAC20] Kirsten H, Al-Hasani H, Holdt L, Gross A, Beutner F, Krohn K, Horn K, Ahnert P, Burkhardt R, Reiche K, 2015 Dissecting the genetics of the human transcriptome identifies novel trait-related *trans*-eQTLs and corroborates the regulatory relevance of non-protein coding loci. Hum Mol Genet 24: 4746–4763. 10.1093/hmg/ddv194 26019233 PMC4512630

[GR238022WHAC21] Krefting J, Andrade-Navarro MA, Ibn-Salem J. 2018 Evolutionary stability of topologically associating domains is associated with conserved gene regulation. BMC Biol 16: 87 10.1186/s12915-018-0556-x 30086749 PMC6091198

[GR238022WHAC22] Kundaje A, Meuleman W, Ernst J, Bilenky M, Yen A, Heravi-Moussavi A, Kheradpour P, Zhang Z, Wang J, Ziller MJ, 2015 Integrative analysis of 111 reference human epigenomes. Nature 518: 317–330. 10.1038/nature14248 25693563 PMC4530010

[GR238022WHAC23] Lazar NH, Nevonen KA, O'Connell B, McCann C, O'Neill RJ, Green RE, Meyer TJ, Okhovat M, Carbone L. 2018 Epigenetic maintenance of topological domains in the highly rearranged gibbon genome. Genome Res 28: 983–997. 10.1101/gr.233874.117 29914971 PMC6028127

[GR238022WHAC024] Li H. 2011 A statistical framework for SNP calling, mutation discovery, association mapping and population genetical parameter estimation from sequencing data. Bioinformatics 27: 2987–2993. 10.1093/bioinformatics/btr509 21903627 PMC3198575

[GR238022WHAC24] Liang S, Tippens ND, Zhou Y, Mort M, Stenson PD, Cooper DN, Yu H. 2017 iRegNet3D: three-dimensional integrated regulatory network for the genomic analysis of coding and non-coding disease mutations. Genome Biol 18: 10 10.1186/s13059-016-1138-2 28100260 PMC5241969

[GR238022WHAC25] Liu Y, Sarkar A, Kheradpour P, Ernst J, Kellis M. 2017 Evidence of reduced recombination rate in human regulatory domains. Genome Biol 18: 193 10.1186/s13059-017-1308-x 29058599 PMC5651596

[GR238022WHAC26] Lu Y, Quan C, Chen H, Bo X, Zhang C. 2017 3DSNP: a database for linking human noncoding SNPs to their three-dimensional interacting genes. Nucleic Acids Res 45: D643–D649. 10.1093/nar/gkw1022 27789693 PMC5210526

[GR238022WHAC27] MacArthur J, Bowler E, Cerezo M, Gil L, Hall P, Hastings E, Junkins H, McMahon A, Milano A, Morales J, 2017 The new NHGRI-EBI Catalog of published genome-wide association studies (GWAS Catalog). Nucleic Acids Res 45: D896–D901. 10.1093/nar/gkw1133 27899670 PMC5210590

[GR238022WHAC28] McKinney W. 2012 Python for data analysis. O'Reilly, Sebastopol, CA.

[GR238022WHAC29] McVean GAT, Myers SR, Hunt S, Deloukas P, Bentley DR, Donnelly P. 2004 The fine-scale structure of recombination rate variation in the human genome. Science 304: 581–584. 10.1126/science.1092500 15105499

[GR238022WHAC30] Mitchel K, Theusch E, Cubitt C, Dosé AC, Stevens K, Naidoo D, Medina MW. 2016 RP1-13D10.2 is a novel modulator of statin-induced changes in cholesterol. Circ Cardiovasc Genet 9: 223–230. 10.1161/CIRCGENETICS.115.001274 27071970 PMC4917428

[GR238022WHAC31] Mumbach MR, Satpathy AT, Boyle EA, Dai C, Gowen BG, Cho SW, Nguyen ML, Rubin AJ, Granja JM, Kazane KR, 2017 Enhancer connectome in primary human cells identifies target genes of disease-associated DNA elements. Nat Genet 49: 1602–1612. 10.1038/ng.3963 28945252 PMC5805393

[GR238022WHAC32] Myers S, Bottolo L, Freeman C, McVean G, Donnelly P. 2005 A fine-scale map of recombination rates and hotspots across the human genome. Science 310: 321–324. 10.1126/science.1117196 16224025

[GR238022WHAC33] Nishizaki SS, Boyle AP. 2017 Mining the unknown: assigning function to noncoding single nucleotide polymorphisms. Trends Genet 33: 34–45. 10.1016/j.tig.2016.10.008 27939749 PMC5553318

[GR238022WHAC34] Ong CT, Corces VG. 2014 CTCF: an architectural protein bridging genome topology and function. Nat Rev Genet 15: 234–246. 10.1038/nrg3663 24614316 PMC4610363

[GR238022WHAC35] Parker SCJ, Stitzel ML, Taylor DL, Orozco JM, Erdos MR, Akiyama JA, van Bueren KL, Chines PS, Narisu N, NISC Comparative Sequencing Program, 2013 Chromatin stretch enhancer states drive cell-specific gene regulation and harbor human disease risk variants. Proc Natl Acad Sci 110: 17921–17926. 10.1073/pnas.1317023110 24127591 PMC3816444

[GR238022WHAC36] Quinlan AR, Hall IM. 2010 BEDTools: a flexible suite of utilities for comparing genomic features. Bioinformatics 26: 841–842. 10.1093/bioinformatics/btq033 20110278 PMC2832824

[GR238022WHAC037] R Core Team. 2018 R: a language and environment for statistical computing. R Foundation for Statistical Computing, Vienna, Austria https://www.R-project.org/.

[GR238022WHAC37] Rao SS, Huntley MH, Durand NC, Stamenova EK, Bochkov ID, Robinson JT, Sanborn AL, Machol I, Omer AD, Lander ES, 2014 A 3D map of the human genome at kilobase resolution reveals principles of chromatin looping. Cell 159: 1665–1680. 10.1016/j.cell.2014.11.021 25497547 PMC5635824

[GR238022WHAC38] Sanjana NE, Wright J, Zheng K, Shalem O, Fontanillas P, Joung J, Cheng C, Regev A, Zhang F. 2016 High-resolution interrogation of functional elements in the noncoding genome. Science 353: 1545–1549. 10.1126/science.aaf7613 27708104 PMC5144102

[GR238022WHAC39] Tange O. 2011 GNU Parallel: the Command-Line power tool. ;login: the USENIX Magazine 36: 42–47.

[GR238022WHAC40] Welter D, MacArthur J, Morales J, Burdett T, Hall P, Junkins H, Klemm A, Flicek P, Manolio T, Hindorff L, 2014 The NHGRI GWAS Catalog, a curated resource of SNP-trait associations. Nucleic Acids Res 42: D1001–D1006. 10.1093/nar/gkt1229 24316577 PMC3965119

[GR238022WHAC41] Wickham H. 2009 ggplot2: elegant graphics for data analysis. Springer-Verlag, New York.

[GR238022WHAC42] Won H, de la Torre-Ubieta L, Stein JL, Parikshak NN, Huang J, Opland CK, Gandal MJ, Sutton GJ, Hormozdiari F, Lu D, 2016 Chromosome conformation elucidates regulatory relationships in developing human brain. Nature 538: 523–527. 10.1038/nature19847 27760116 PMC5358922

