# Supplementary material for: Most chromatin interactions are not in linkage disequilibrium
Source: Genome Res. 2019 Mar;29(3):334–43. doi: 10.1101/gr.238022.118 (PMC6396425; doi:10.1101/gr.238022.118)
Supplement: Supplemental Material [file supp_29_3_334__index.html]

Most chromatin interactions are not in linkage disequilibrium — Most chromatin interactions are not in linkage disequilibrium — Supplemental Material 

# Most chromatin interactions are not in linkage disequilibrium

## Supplemental Material

- Supplemental\_Fig\_S1.pdf
- Supplemental\_Fig\_S2.pdf
- Supplemental\_Fig\_S3.pdf
- Supplemental\_Fig\_S4.pdf
- Supplemental\_Fig\_S5.pdf
- Supplemental\_Fig\_S6.pdf
- Supplemental\_Fig\_S7.pdf
- Supplemental\_Fig\_S8.pdf
- Supplemental\_Fig\_S9.pdf
- Supplemental\_Table\_S1.pdf
- Supplemental\_Table\_S2.pdf
- Supplemental\_code.zip
