# Supplementary material for: Most chromatin interactions are not in linkage disequilibrium
Source: Genome Res. 2019 Mar;29(3):334–43. doi: 10.1101/gr.238022.118 (PMC6396425; doi:10.1101/gr.238022.118)
Supplement: Supplemental Material [file supp_gr.238022.118_Supplemental_Fig_S1.pdf]

(a)

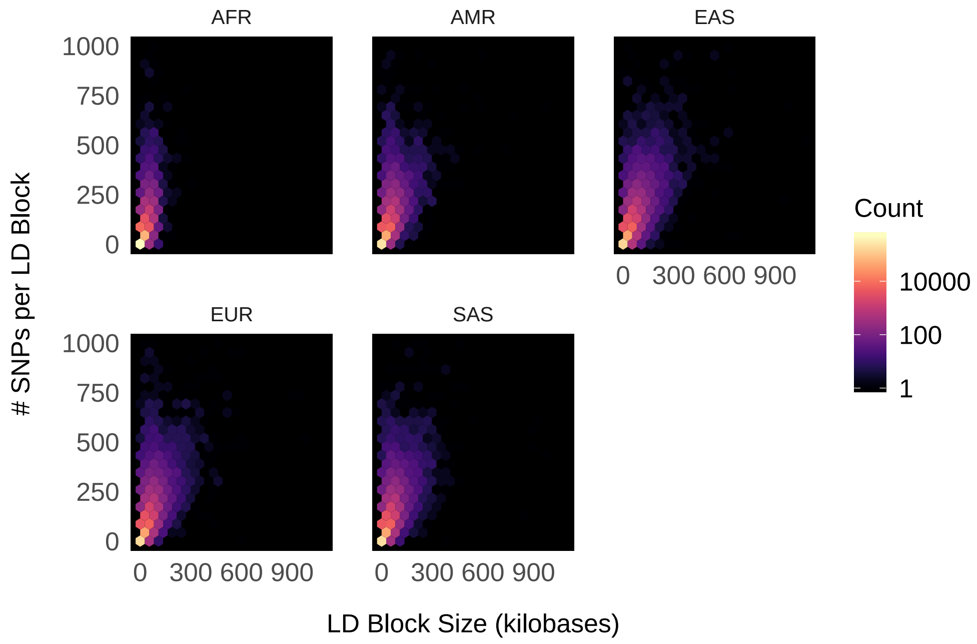

(b)

Super Pop.	LD Block Size Bin (kb)	Median # SNPs
AFR	[0.002, 0.239)	2
AFR	[0.239, 1.108)	3
AFR	[1.108, 3.987)	8
AFR	[3.987,289.270]	27
AMR	[0.002, 0.359)	2
AMR	[0.359, 2.075)	4
AMR	[2.075, 7.773)	11
AMR	[7.773,967.830]	40
EAS	[0.002, 0.422)	2
EAS	[0.422, 2.831)	4
EAS	[2.831, 11.010)	14
EAS	[11.010,1084.750]	51
EUR	[0.002, 0.39)	2
EUR	[0.390, 2.50)	4
EUR	[2.500, 9.71)	13
EUR	[9.713,973.50]	49
SAS	[0.002, 0.369)	2
SAS	[0.369, 2.277)	4
SAS	[2.277, 8.784)	13
SAS	[8.784,974.911]	46

Supplemental Figure 1. The number of SNPs in an LD block scales with its length (kb, quartile bins). Most SNPs are in LD blocks only several kb in length, though some are in much longer blocks along with dozens or even hundreds of other SNPs.
