# Supplementary material for: Most chromatin interactions are not in linkage disequilibrium
Source: Genome Res. 2019 Mar;29(3):334–43. doi: 10.1101/gr.238022.118 (PMC6396425; doi:10.1101/gr.238022.118)
Supplement: Supplemental Material [file supp_gr.238022.118_Supplemental_Fig_S2.pdf]

(a) GM12878, EAS, chr14:85440381-93881268 (8.5Mb)

(b) GM12878, EAS, chr14:88440381-88881268 (400kb)

(c) NHEK, EAS, chr14:88440381-88881268 (400kb)

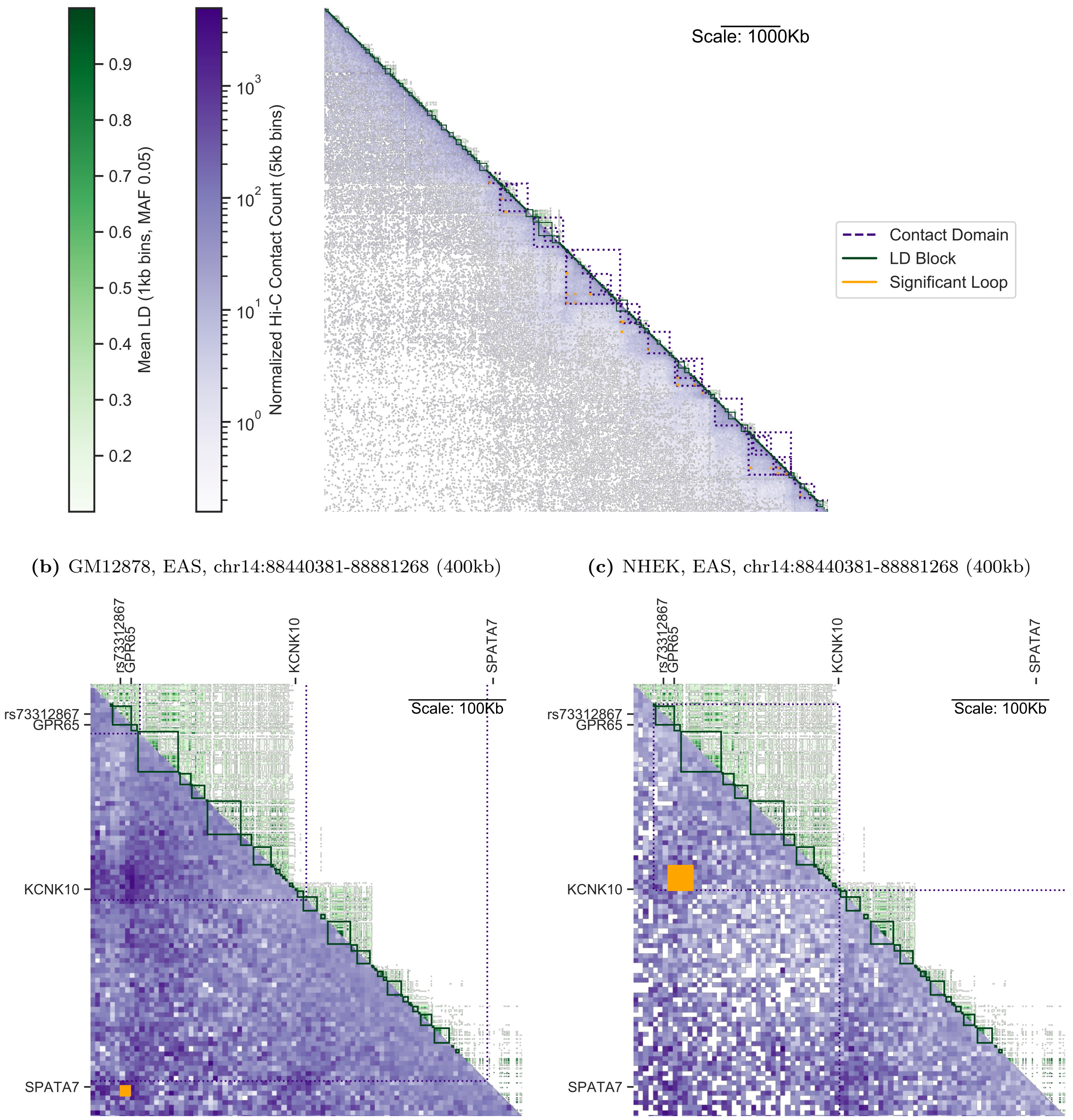

Supplemental Figure 2. An annotated matrix illustrates differences between the genomic scales of LD (The 1000 Genomes Project Consortium 2015) (R^2, upper triangle, green) versus Hi-C contact frequency (Rao et al. 2014) (lower triangle, purple). Rows and columns are binned genomic coordinates (hg19) with lower bins near the upper left; for example, row 10 column 11 stores the LD between a bin and its neighbor, while row 11 column 10 stores the contact frequency. More frequent contacts (5kb bins) are darker purple; higher LD (averaged over non-zero LD pairs in 1kb bins) are darker green. Contact domains (nested purple squares) and significant interactions (orange squares) were computed from Hi-C data. *(A)* A representative 8.5Mb locus on chromosome 14 shows Hi-C contacts (GM12878 cells) span much longer distances than LD (EAS super-population). *(B)* A 400kb locus on the same chromosome illustrates the complexities of mapping a non-coding SNP (rs73312867) to a target gene. The closest gene *GPR65* falls within the same LD block as the SNP. However, Hi-C data shows the SNP contacts the *SPATA7* gene ≈ 380kb away, skipping over intervening gene *KCNK10*. *(C)* In NHEK cells, the SNP interacts with *KCNK10* instead.
