# Supplementary material for: Most chromatin interactions are not in linkage disequilibrium
Source: Genome Res. 2019 Mar;29(3):334–43. doi: 10.1101/gr.238022.118 (PMC6396425; doi:10.1101/gr.238022.118)
Supplement: Supplemental Material [file supp_gr.238022.118_Supplemental_Fig_S3.pdf]

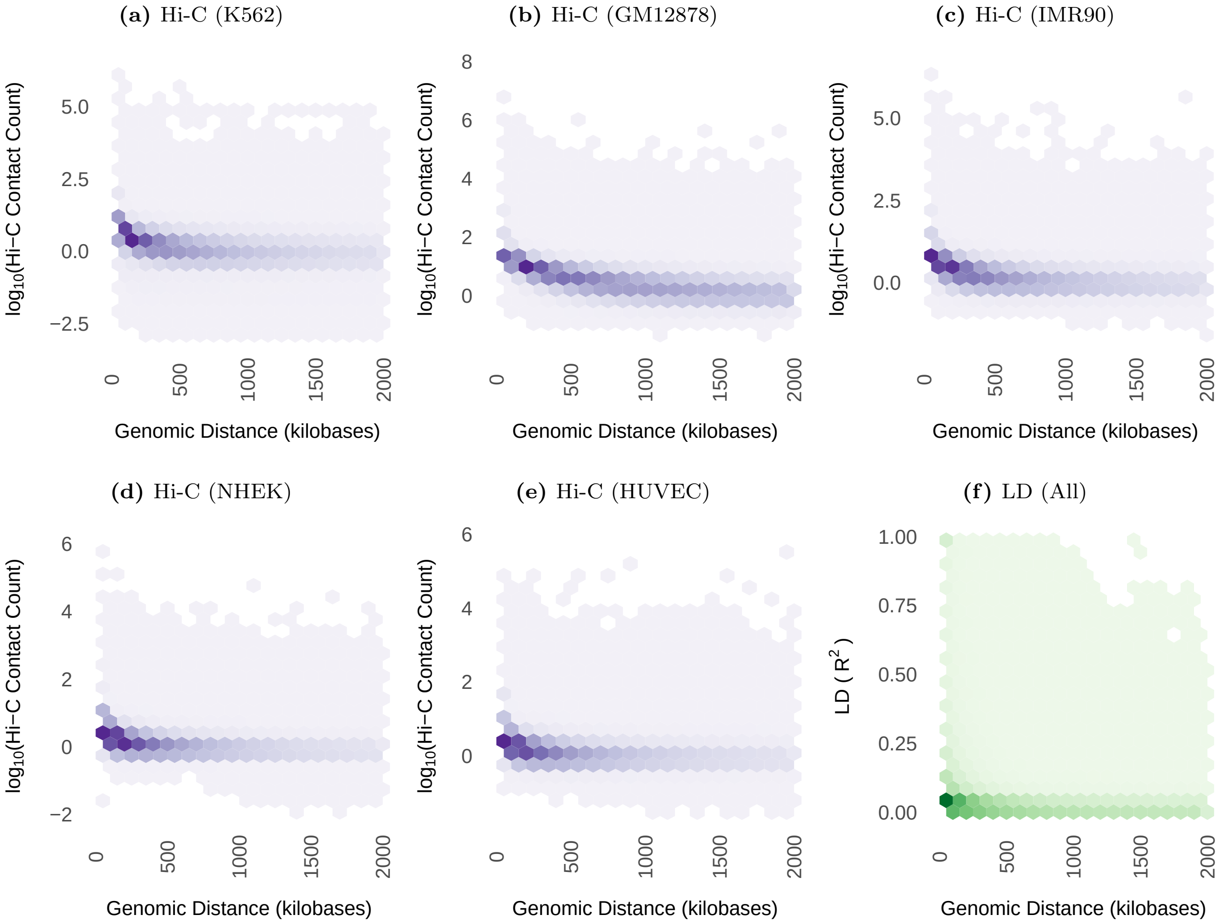

Supplemental Figure 3. Both Hi-C contact frequency (Rao et al. 2014) *(A-E)* and *(F)* LD are anti-correlated with genomic distance (Spearman ρ between -0.5 and -0.71 for Hi-C across cell lines; $\rho \approx -0.52$ for LD). All plots display non-zero values from their respective datasets. LD decays towards zero at much shorter genomic distance than contact frequency, with most high LD SNP pairs concentrated below 50kb. Hi-C contacts are common at longer genomic distances up to and exceeding the median length of contact domains (250kb) or TADs (840kb). Supplemental Figure 4 shows nearly identical LD scaling per super-population. *(F)* 836 million biallelic SNP pairs on chromosome 14, representative of other chromosomes.
