# Supplementary material for: Most chromatin interactions are not in linkage disequilibrium
Source: Genome Res. 2019 Mar;29(3):334–43. doi: 10.1101/gr.238022.118 (PMC6396425; doi:10.1101/gr.238022.118)
Supplement: Supplemental Material [file supp_gr.238022.118_Supplemental_Fig_S5.pdf]

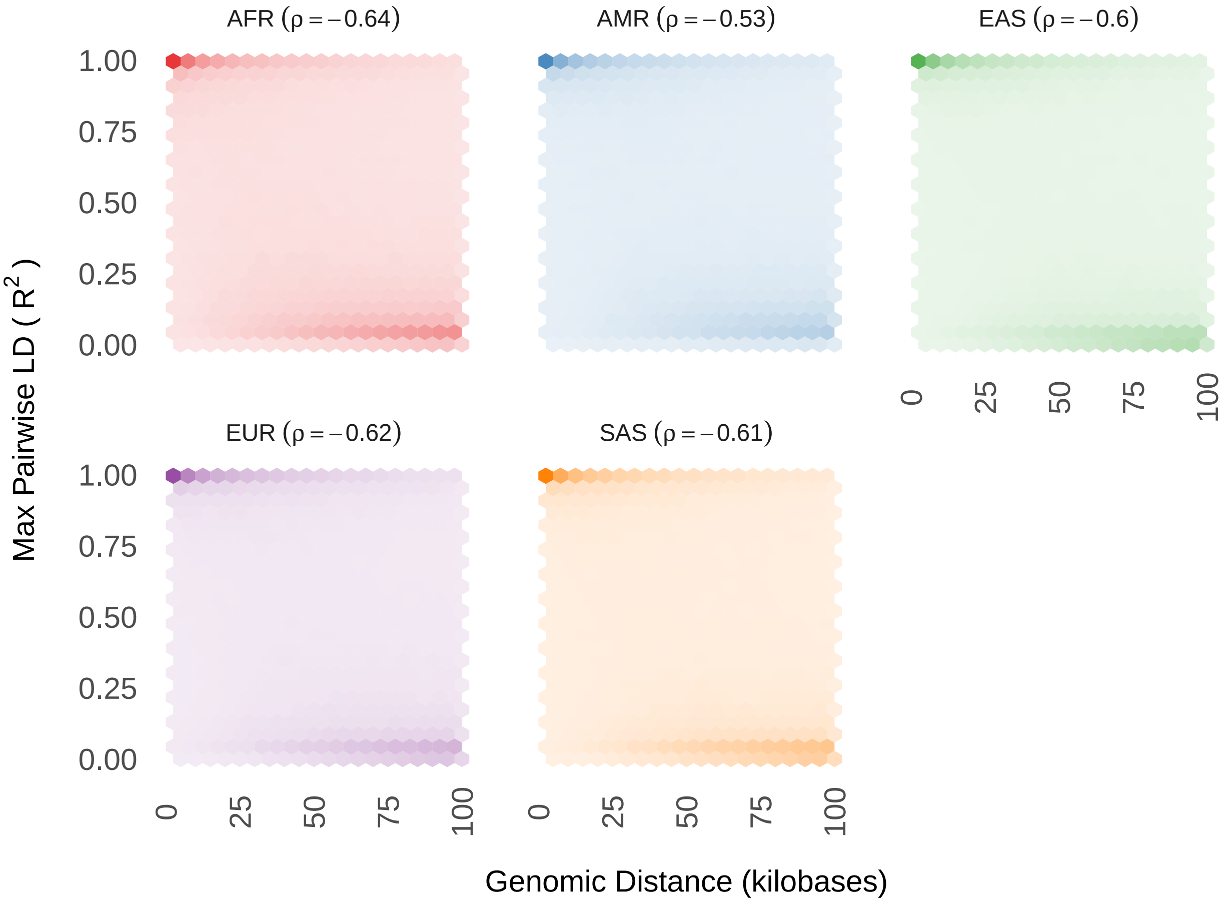

Supplemental Figure 5. Scaling of LD with genomic distance for SNPs located on statistically significant chromatin interactions shows moderate anti-correlation for all super-populations. Figure 3 shows combined LD scaling (not restricted to interacting chromatin) and observed Hi-C contact frequency scaling by cell line.
