# Supplementary figures and images for: Most chromatin interactions are not in linkage disequilibrium
Source: Genome Res. 2019 Mar;29(3):334–43. doi: 10.1101/gr.238022.118 (PMC6396425; doi:10.1101/gr.238022.118)

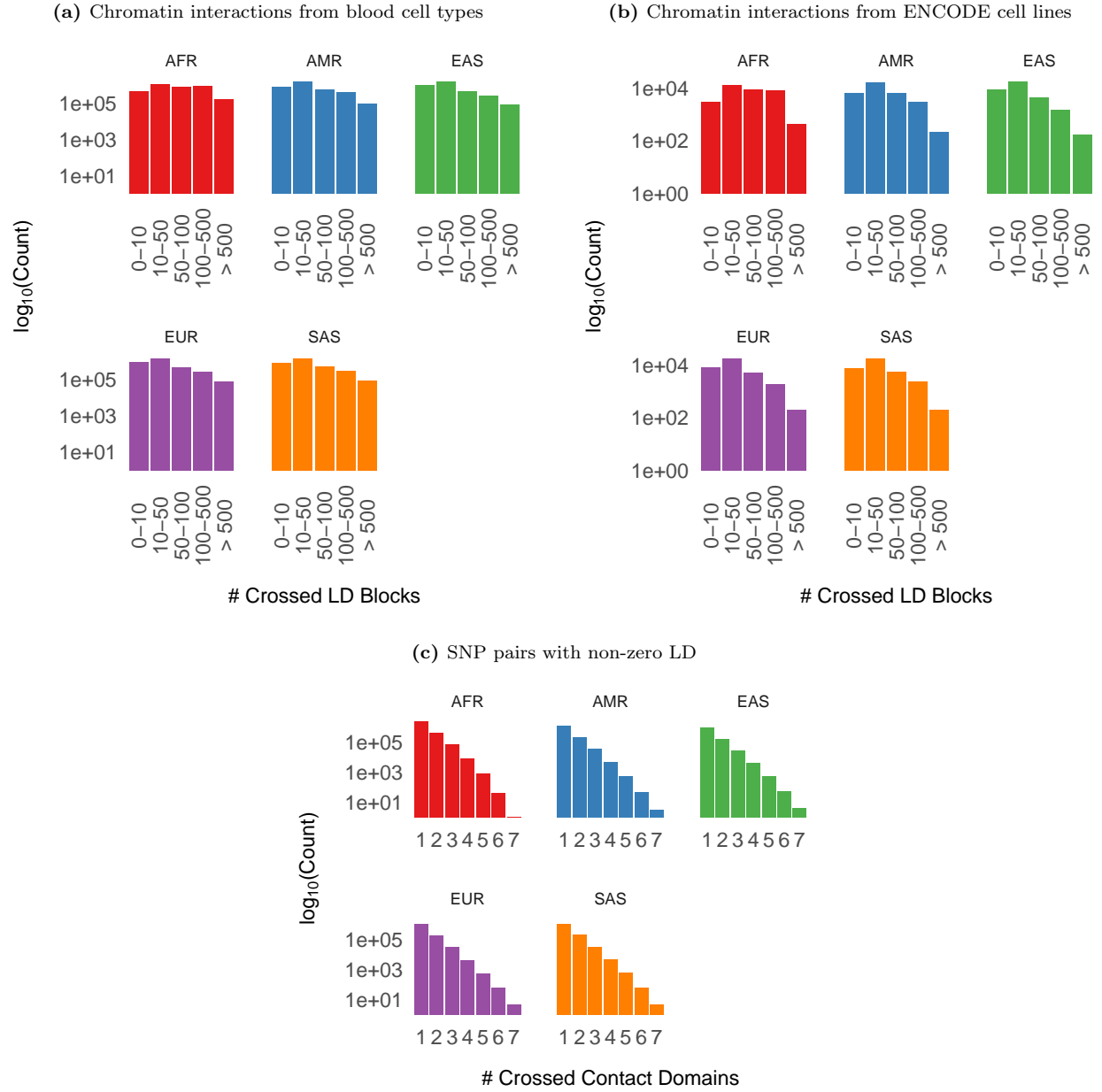

Supplement: Supplemental Material [file supp_gr.238022.118_Supplemental_Fig_S6.pdf]
