# Supplementary material for: Most chromatin interactions are not in linkage disequilibrium
Source: Genome Res. 2019 Mar;29(3):334–43. doi: 10.1101/gr.238022.118 (PMC6396425; doi:10.1101/gr.238022.118)
Supplement: Supplemental Material [file supp_gr.238022.118_Supplemental_Fig_S7.pdf]

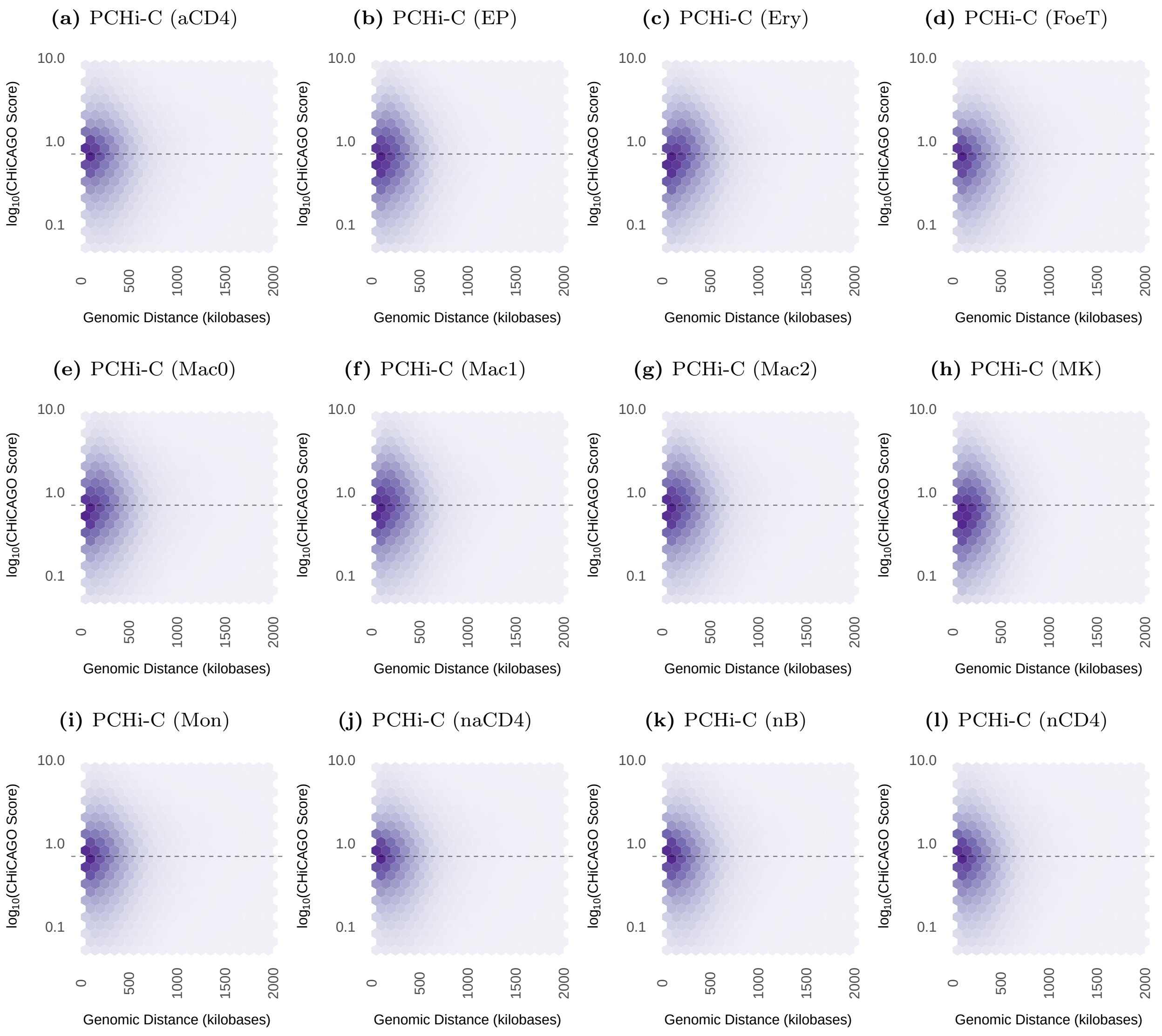

Supplemental Figure 7. Promoter Capture Hi-C contacts are anti-correlated with distance in 17 blood cell types. Decay is similar to Supplemental Figure 3, though here an interaction score (quantified by CHiCAGO, Cairns et al. 2016) is used rather than normalized contact frequency. A score above 5 denotes statistically significant interactions, and this threshold is indicated with a dashed line. Interaction distances are concentrated below 1Mb, though a large number of significant interactions exist at 500kb, well beyond the 40kb drop-off previously observed.

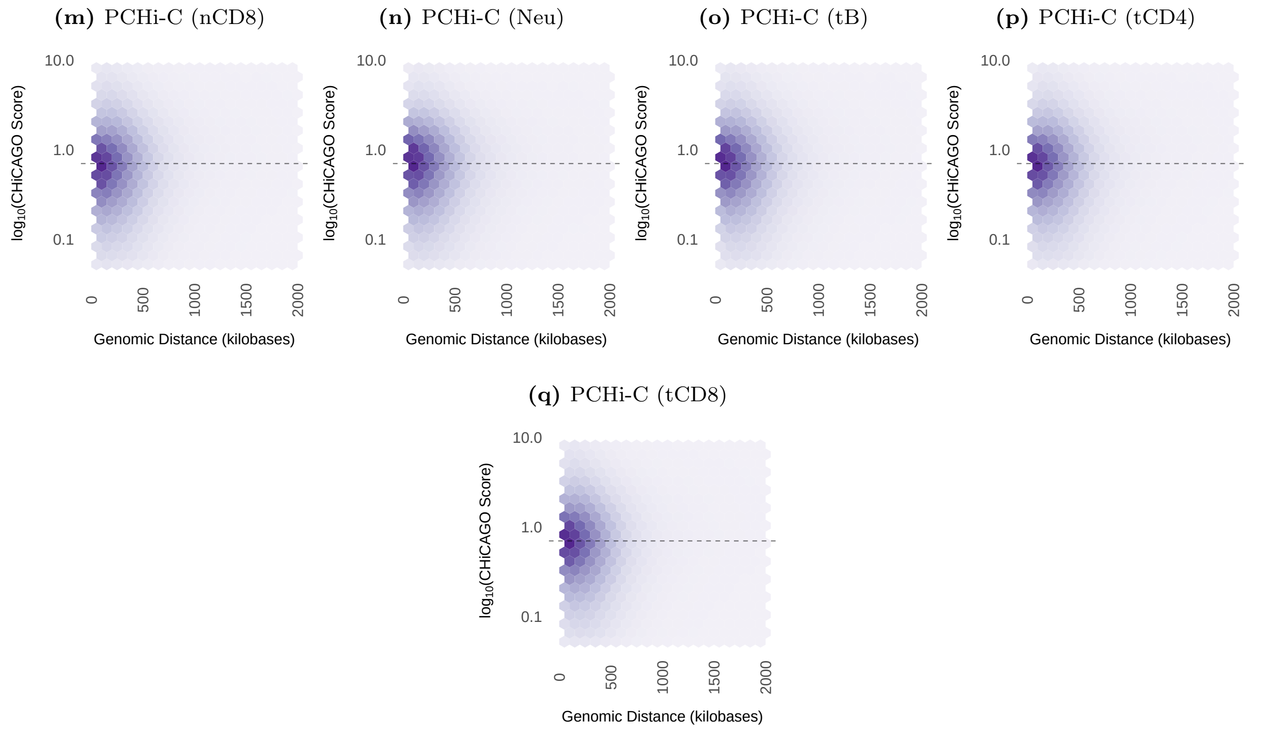

Supplemental Figure 7. Promoter Capture Hi-C contacts are anti-correlated with distance in 17 blood cell types. Decay is similar to Supplemental Figure 3, though here an interaction score (quantified by CHiCAGO, Cairns et al. 2016) is used rather than normalized contact frequency. A score above 5 denotes statistically significant interactions, and this threshold is indicated with a dashed line. Interaction distances are concentrated below 1Mb, though a large number of significant interactions exist at 500kb, well beyond the 40kb drop-off previously observed.
