# Supplementary material for: Most chromatin interactions are not in linkage disequilibrium
Source: Genome Res. 2019 Mar;29(3):334–43. doi: 10.1101/gr.238022.118 (PMC6396425; doi:10.1101/gr.238022.118)
Supplement: Supplemental Material [file supp_gr.238022.118_Supplemental_Fig_S8.pdf]

(a) LD Blocks to Contact Domains

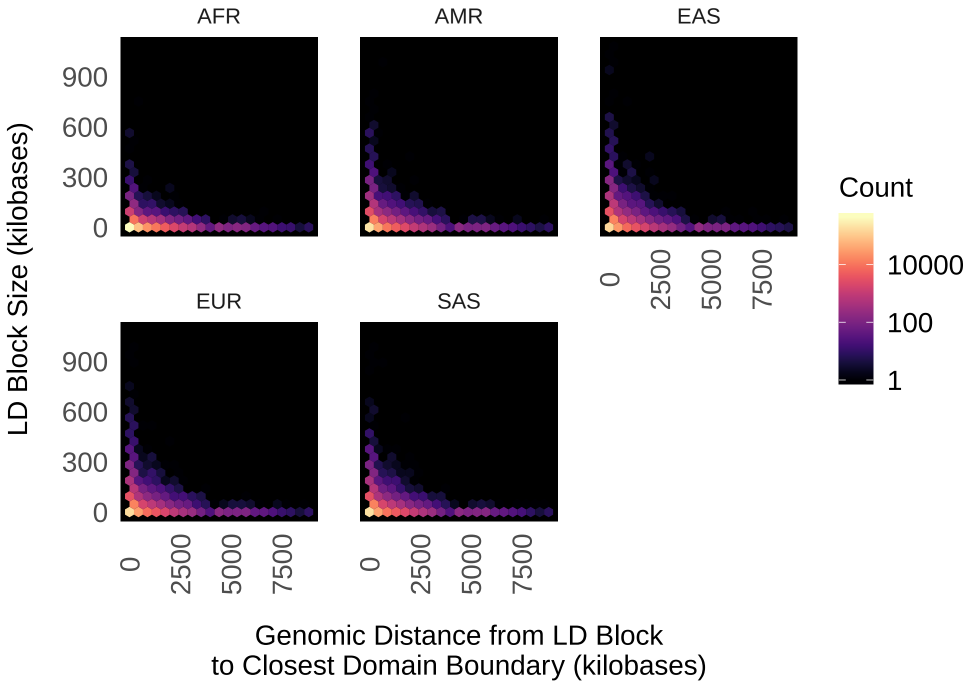

(b) LD Blocks to Contact Domains (zoomed)

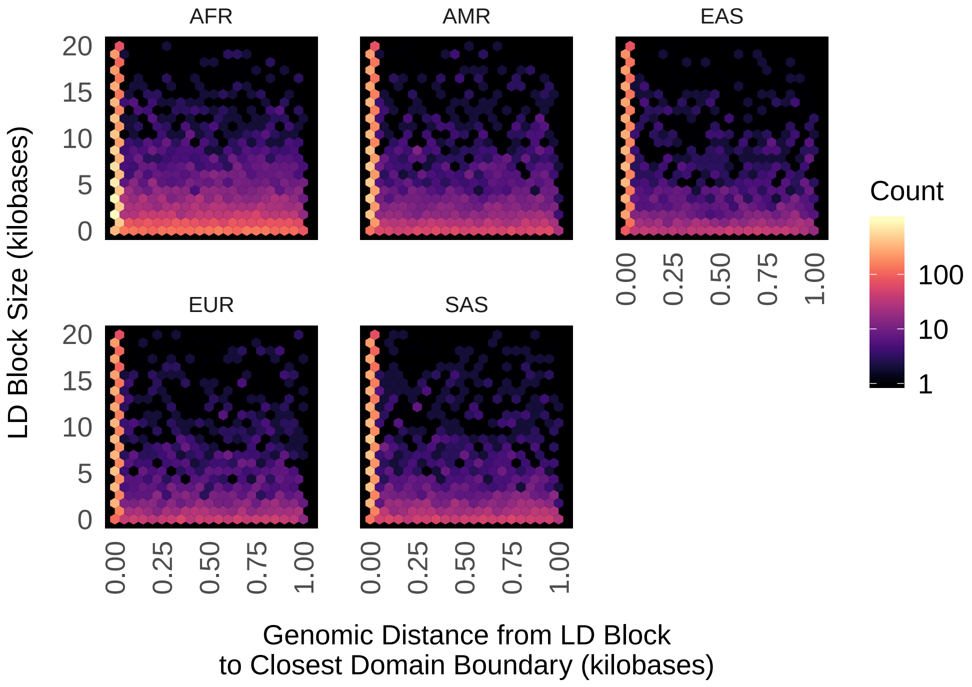

(c) Contact Domains to LD Blocks

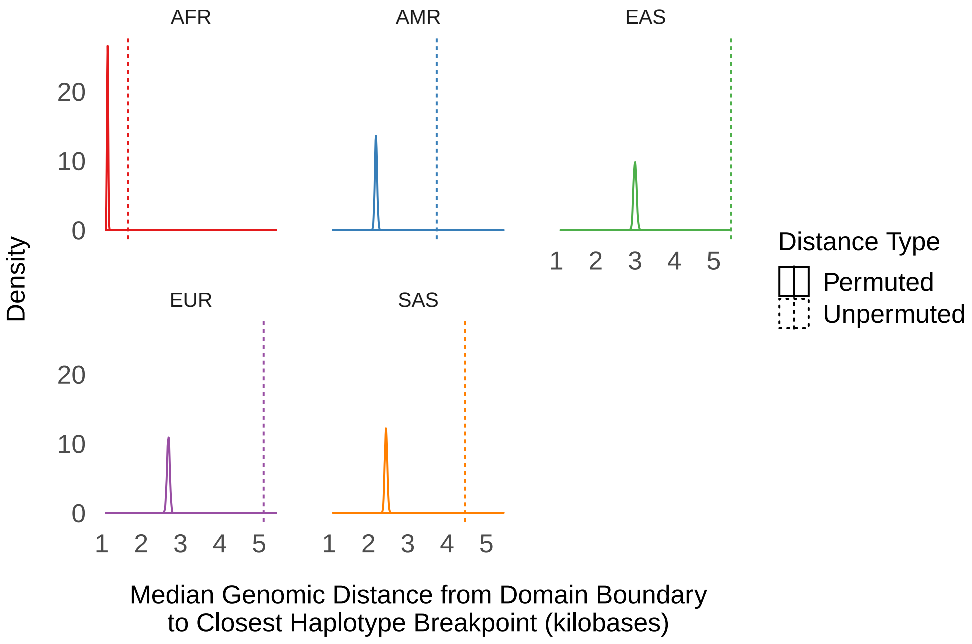

Supplemental Figure 8. *(A)* The size of LD blocks as a function of distance to the closest contact domain (GM12878). Block sizes increase as they approach domain boundaries, with order-of-magnitude increases for blocks that overlap domain boundaries (distance = 0). *(B)* The magnitude of increase is apparent at the kilobase scale. *(C)* The distance from domain boundaries to the nearest LD block boundary, showing the distance distribution for 1000 permutations of LD block locations compared to the non-permuted distance. For all super-populations, LD block boundaries are further away from contact domain boundaries than expected by chance. Combined, this suggests recombination tends to avoid contact domain boundaries.
