# Supplementary material for: Most chromatin interactions are not in linkage disequilibrium
Source: Genome Res. 2019 Mar;29(3):334–43. doi: 10.1101/gr.238022.118 (PMC6396425; doi:10.1101/gr.238022.118)
Supplement: Supplemental Material [file supp_gr.238022.118_Supplemental_Fig_S9.pdf]

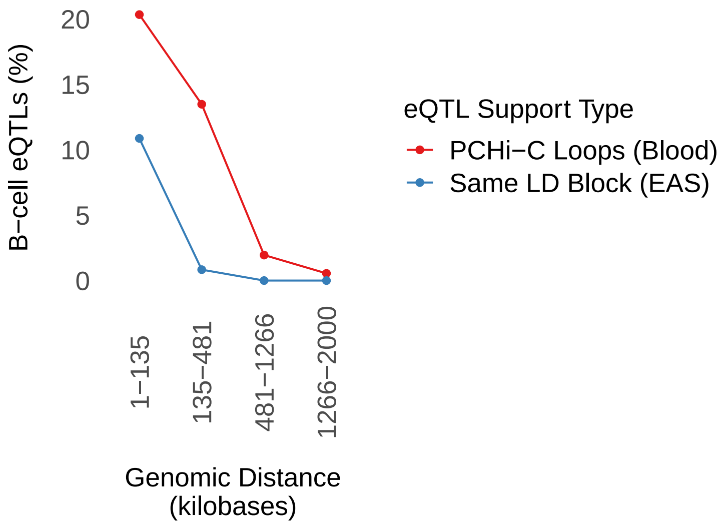

Supplemental Figure 9. Percent of B-cell eQTLs (Fairfax et al. 2012) supported by B-cell PCHi-C interactions (Javierre et al. 2016), compared to eQTLs in the same LD block with their target gene (EAS super-population).
