# Supplementary material for: Most chromatin interactions are not in linkage disequilibrium
Source: Genome Res. 2019 Mar;29(3):334–43. doi: 10.1101/gr.238022.118 (PMC6396425; doi:10.1101/gr.238022.118)
Supplement: Supplemental Material [file supp_gr.238022.118_Supplemental_Table_S1.pdf]

(a) Cell Type Statistics

Assay	Cell Type	Acronym	Reps.	Unique Read Pairs	Sig. Interactions
Hi-C	Lymphoblastoid cells	GM12878	9	4,907,147,001	9,448
Hi-C	Fetal lung fibroblasts	IMR90	2	1,136,673,290	8,040
Hi-C	Epidermal skin keratinocytes	NHEK	1	664,899,299	4,929
Hi-C	Erythroleukemia cells	K562	2	932,208,867	6,057
Hi-C	Umbilical vein endothelial cells	HUVEC	1	460,393,495	3,865
PCHi-C	Megakaryocytes	MK	4	653,848,788	150,779
PCHi-C	Erythroblasts	Ery	3	588,786,672	151,215
PCHi-C	Neutrophils	Neu	3	736,055,569	142,435
PCHi-C	Monocytes	Mon	3	572,357,387	165,947
PCHi-C	Macrophages M0	Mac0	3	668,675,248	180,190
PCHi-C	Macrophages M1	Mac1	3	497,683,496	171,031
PCHi-C	Macrophages M2	Mac2	3	523,561,551	186,172
PCHi-C	Endothelial precursors	EP	3	420,536,621	145,888
PCHi-C	Naive B cells	nB	3	629,928,642	189,720
PCHi-C	Total B cells	tB	3	702,533,922	213,539
PCHi-C	Fetal thymus	FoeT	3	776,491,344	166,743
PCHi-C	Naive $CD4^+$ T cells	nCD4	4	844,697,853	210,074
PCHi-C	Total $CD4^+$ T cells	tCD4	3	836,974,777	199,525
PCHi-C	Non-activated total $CD4^+$ T cells	naCD4	3	721,030,702	211,720
PCHi-C	Activated total $CD4^+$ T cells	aCD4	3	749,720,649	213,235
PCHi-C	Naive $CD8^+$ T cells	nCD8	3	747,834,572	216,232
PCHi-C	Total $CD8^+$ T cells	tCD8	3	628,771,947	204,382

(b) LD Statistics

Superpop.	# SNP Pairs	# LD Blocks	BL .05	BL .5	BL .95	BP .05	BP .5	BP .95
AFR	5,447,813,153	565,469	10	1,106	16,540	2	5	53
AMR	10,353,472,856	296,861	9	2,076	33,784	2	7	81
EAS	3,166,821,307	216,961	8	2,833	47,733	2	8	105
EUR	4,156,780,073	242,446	8	2,502	43,067	2	7	100
SAS	4,079,746,085	266,508	9	2,277	38,378	2	7	93

Supplemental Table 1. *(A)* Summary of Hi-C (Rao et al. 2014) and promoter capture Hi-C (PCHi-C, Javierre et al. 2016) datasets. Statistically significant (sig.) Hi-C interactions were called on combined replicates (reps.) by the Juicer pipeline (Durand et al. 2016) with a 10% false discovery rate, while PCHi-C interactions were scored by the CHiCAGO pipeline (Cairns et al. 2016) with a significance threshold of 5. PCHi-C has 15-17 fold enrichment for promoter interactions, resulting in an effective coverage of 165 billion read pairs compared to 15 billion for Hi-C (Javierre et al. 2016). *(B)* Number of unique SNP pairs and LD blocks per super-population. LD block quantiles (.05, .5, and .95) for length (BL) and unique SNP pairs (BP) are also given. SNPs were filtered as described in Methods.
