# Supplementary material for: Most chromatin interactions are not in linkage disequilibrium
Source: Genome Res. 2019 Mar;29(3):334–43. doi: 10.1101/gr.238022.118 (PMC6396425; doi:10.1101/gr.238022.118)
Supplement: Supplemental Material [file supp_gr.238022.118_Supplemental_Table_S2.pdf]

Cell Type	Superpopulation	Significant	mean(max(R^2))
K562	AFR	0	0.193
		1	0.163
	AMR	0	0.242
		1	0.216
	EAS	0	0.163
		1	0.137
	EUR	0	0.191
		1	0.162
	SAS	0	0.172
		1	0.144
GM12878	AFR	0	0.161
		1	0.141
	AMR	0	0.202
		1	0.183
	EAS	0	0.145
		1	0.123
	EUR	0	0.167
		1	0.145
	SAS	0	0.152
		1	0.129
IMR90	AFR	0	0.198
		1	0.165
	AMR	0	0.242
		1	0.209
	EAS	0	0.177
		1	0.145
	EUR	0	0.203
		1	0.169
	SAS	0	0.186
		1	0.153
NHEK	AFR	0	0.216
		1	0.175
	AMR	0	0.267
		1	0.233
	EAS	0	0.167
		1	0.133
	EUR	0	0.200
		1	0.166
	SAS	0	0.177
		1	0.142
HUVEC	AFR	0	0.209
		1	0.176
	AMR	0	0.260
		1	0.230
	EAS	0	0.166
		1	0.139
	EUR	0	0.199
		1	0.169
	SAS	0	0.178

Continued on next page

Cell Type	Superpopulation	Significant	mean(max(R^2))
		1	0.149
Mon	AFR	0	0.151
		1	0.145
	AMR	0	0.192
		1	0.183
	EAS	0	0.149
		1	0.145
	EUR	0	0.164
		1	0.158
	SAS	0	0.152
		1	0.146
Mac0	AFR	0	0.142
		1	0.124
	AMR	0	0.183
		1	0.160
	EAS	0	0.139
		1	0.122
	EUR	0	0.155
		1	0.136
	SAS	0	0.143
		1	0.124
Mac1	AFR	0	0.147
		1	0.134
	AMR	0	0.188
		1	0.171
	EAS	0	0.144
		1	0.133
	EUR	0	0.160
		1	0.146
	SAS	0	0.148
		1	0.136
Mac2	AFR	0	0.146
		1	0.141
	AMR	0	0.187
		1	0.178
	EAS	0	0.144
		1	0.139
	EUR	0	0.159
		1	0.154
	SAS	0	0.147
		1	0.142
Neu	AFR	0	0.157
		1	0.174
	AMR	0	0.198
		1	0.212
	EAS	0	0.155
		1	0.170
	EUR	0	0.171
		1	0.184

Continued on next page

Cell Type	Superpopulation	Significant	mean(max(R^2))
	SAS	0	0.159
		1	0.171
MK	AFR	0	0.160
		1	0.162
	AMR	0	0.202
		1	0.200
	EAS	0	0.159
		1	0.163
	EUR	0	0.175
		1	0.177
	SAS	0	0.162
		1	0.165
EP	AFR	0	0.148
		1	0.141
	AMR	0	0.190
		1	0.177
	EAS	0	0.145
		1	0.140
	EUR	0	0.161
		1	0.154
	SAS	0	0.148
		1	0.144
Ery	AFR	0	0.148
		1	0.162
	AMR	0	0.189
		1	0.199
	EAS	0	0.146
		1	0.161
	EUR	0	0.162
		1	0.177
	SAS	0	0.149
		1	0.163
FoeT	AFR	0	0.128
		1	0.131
	AMR	0	0.168
		1	0.169
	EAS	0	0.125
		1	0.132
	EUR	0	0.139
		1	0.144
	SAS	0	0.128
		1	0.133
nCD4	AFR	0	0.149
		1	0.151
	AMR	0	0.191
		1	0.190
	EAS	0	0.148
		1	0.153
	EUR	0	0.163

Continued on next page

Cell Type	Superpopulation	Significant	mean(max(R^2))
		1	0.166
	SAS	0	0.151
		1	0.155
tCD4	AFR	0	0.139
		1	0.141
	AMR	0	0.179
		1	0.180
	EAS	0	0.137
		1	0.143
	EUR	0	0.151
		1	0.156
	SAS	0	0.140
		1	0.144
aCD4	AFR	0	0.151
		1	0.151
	AMR	0	0.192
		1	0.191
	EAS	0	0.149
		1	0.154
	EUR	0	0.165
		1	0.168
	SAS	0	0.152
		1	0.155
naCD4	AFR	0	0.145
		1	0.145
	AMR	0	0.187
		1	0.184
	EAS	0	0.144
		1	0.147
	EUR	0	0.159
		1	0.161
	SAS	0	0.147
		1	0.148
nCD8	AFR	0	0.146
		1	0.151
	AMR	0	0.187
		1	0.190
	EAS	0	0.144
		1	0.153
	EUR	0	0.159
		1	0.165
	SAS	0	0.147
		1	0.154
tCD8	AFR	0	0.153
		1	0.155
	AMR	0	0.195
		1	0.194
	EAS	0	0.153
		1	0.160

Continued on next page

Cell Type	Superpopulation	Significant	mean(max(R^2))
	EUR	0	0.168
		1	0.172
	SAS	0	0.156
		1	0.160
nB	AFR	0	0.145
		1	0.142
	AMR	0	0.187
		1	0.180
	EAS	0	0.144
		1	0.144
	EUR	0	0.159
		1	0.157
	SAS	0	0.146
		1	0.145
tB	AFR	0	0.143
		1	0.141
	AMR	0	0.184
		1	0.178
	EAS	0	0.141
		1	0.143
	EUR	0	0.156
		1	0.155
	SAS	0	0.144
		1	0.144

Supplemental Table 2. The maximum pairwise LD between SNPs was computed for SNPs located on the fragments of statistically significant and distance-matched non-significant chromatin interactions (*interaction LD*) in 5 Hi-C and 17 PCHi-C datasets. The mean interaction LD per cell type is given for statistically significant (1) and non-significant (0) interactions.
